# Public health and economic benefits of seasonal influenza vaccination in risk groups in France, Italy, Spain and the UK: state of play and perspectives

**DOI:** 10.1186/s12889-024-18694-5

**Published:** 2024-05-03

**Authors:** Thierry Rigoine de Fougerolles, Théophile Baïssas, Guillaume Perquier, Olivier Vitoux, Pascal Crépey, José Bartelt-Hofer, Hélène Bricout, Audrey Petitjean

**Affiliations:** 1 Corporate Value Associates, Paris, France; 2 Corporate Value Associates, London, UK; 3 Univ Rennes, EHESP, CNRS, INSERM, Arènes - UMR 6051, RSMS – U 1309, Rennes, France; 4 Sanofi, 14, Espace Henry Vallée, 69007 Lyon, France

**Keywords:** Influenza, Public health policy, Vaccines and immunisation, Vaccination coverage rate, Modelling, Epidemiology, Influenza burden, Economic impact

## Abstract

**Background:**

Seasonal influenza epidemics have a substantial public health and economic burden, which can be alleviated through vaccination. The World Health Organization (WHO) recommends a 75% vaccination coverage rate (VCR) in: older adults (aged ≥ 65 years), individuals with chronic conditions, pregnant women, children aged 6–24 months and healthcare workers. However, no European country achieves this target in all risk groups. In this study, potential public health and economic benefits achieved by reaching 75% influenza VCR was estimated in risk groups across four European countries: France, Italy, Spain, and the UK.

**Methods:**

A static epidemiological model was used to estimate the averted public health and economic burden of increasing the 2021/2022 season VCR to 75%, using the efficacy data of standard-dose quadrivalent influenza vaccine. For each country and risk group, the most recent data on population size, VCR, pre-pandemic influenza epidemiology, direct medical costs and absenteeism were identified through a systematic literature review, supplemented by manual searching. Outcomes were: averted influenza cases, general practitioner (GP) visits, hospitalisations, case fatalities, number of days of work lost, direct medical costs and absenteeism-related costs.

**Results:**

As of the 2021/2022 season, the UK achieved the highest weighted VCR across risk groups (65%), followed by Spain (47%), France (44%) and Italy (44%). Based on modelling, the 2021/2022 VCR prevented an estimated 1.9 million influenza cases, avoiding 375,200 GP visits, 73,200 hospitalisations and 38,400 deaths. To achieve the WHO 75% VCR target, an additional 24 million at-risk individuals would need to be vaccinated, most of which being older adults and patients with chronic conditions. It was estimated that this could avoid a further 918,200 influenza cases, 332,000 GP visits, 16,300 hospitalisations and 6,300 deaths across the four countries, with older adults accounting for 52% of hospitalisations and 80% of deaths. An additional €84 million in direct medical costs and €79 million in absenteeism costs would be saved in total, with most economic benefits delivered in France.

**Conclusions:**

Older adults represent most vaccine-preventable influenza cases and deaths, followed by individuals with chronic conditions. Health authorities should prioritise vaccinating these populations for maximum public health and economic benefits.

**Supplementary Information:**

The online version contains supplementary material available at 10.1186/s12889-024-18694-5.

## Background

Seasonal influenza affects 5–10% of the global population [1], accounting for 290,000–650,000 annual deaths globally [2, 3], not including secondary complications or underlying conditions exacerbated by influenza [3]. In addition, a 2018 systematic review of randomised controlled trials designed to determine the incidence of influenza showed that 1 in 10 unvaccinated adults and 1 in 5 unvaccinated children were infected with influenza annually [4]. Risk groups for severe influenza include individuals with chronic conditions (such as human immunodeficiency viruses [HIV]/acquired immunodeficiency syndrome [AIDS], asthma, chronic heart or lung diseases), older adults (typically those aged ≥ 65 years), pregnant women, and young children aged 6–24 months [5]. Healthcare workers (HCW) also comprise a risk group, being at increased personal risk of exposure to infection and a potential source of further transmission [6].

Increased general practitioner (GP) visits, hospitalisations, and deaths related to influenza infection are especially common in adults aged ≥ 65 years and in individuals with chronic conditions [7]. In addition, pregnancy is associated with elevated risk of influenza-related death and intensive care unit admission [8]. The incidence of influenza-related complications leading to hospitalisation also increases in at-risk individuals compared with individuals not at risk [9]. Of those hospitalised, approximately 10% will be defined as complicated hospitalisations, which require mechanical ventilation support, lead to intensive care unit admission, or result in death [10]. Complicated hospitalisations contribute substantially to the overall influenza-related healthcare burden due to excess consultations and hospitalisation costs, as well as the broader societal and economic burden associated with reduced productivity [11–13].

Vaccination against seasonal influenza is effective in reducing both influenza disease burden in risk groups and the cost of annual influenza epidemics [13]. In 2003, the World Health Organization (WHO) urged European Union (EU) and European Economic Area member states to achieve a 75% vaccination coverage rate (VCR) target among risk groups by 2010 [14, 15]. Despite this target, VCRs in most countries across Europe remained suboptimal in all risk groups during the 2022‒2023 influenza season [16]. As such, the WHO-recommended 75% target VCR remains unchanged. To appropriately allocate resources, understanding the public heath, economic, and broader benefits of vaccination is required; this can be accomplished by measuring achieved VCR and modelling the impact of increasing VCR [17]. Although such analyses have proven beneficial in decision-making around the use of vaccine prioritisation strategies [18], no up-to-date analyses have measured the benefits of increasing the influenza VCR in Europe. The potential public health and economic benefits of reaching a target seasonal influenza VCR of 100% for all risk groups across 25 EU member states have been estimated in 2006 [13]. Achieving such a target would have led to an estimated approximate reduction in influenza cases of 7.22 million, 797,000 fewer hospital admissions and 68,500 fewer influenza related deaths for all 25 EU member states [13]. A subsequent 2014 study, using an adapted version of the 2006 model [17], estimated that achieving 75% VCR across 27 EU member states would increase the number of averted annual cases of influenza by 1.6–1.7 million and would prevent influenza-related costs of between €190 and €226 million. Updated data are needed to provide accurate estimates of the potential current health and economic benefits, along with a need for data that focus on the potential benefits in groups at risk from severe influenza.

This study aimed to provide estimates of the health and economic benefits associated with seasonal influenza immunisation at the 2021/2022 VCR in France, Italy, Spain and the UK, while exploring the potential further benefits achieved by reaching the WHO-recommended 75% VCR target in risk groups in these countries.

## Methods

### Computational model

**Fig. 1:**
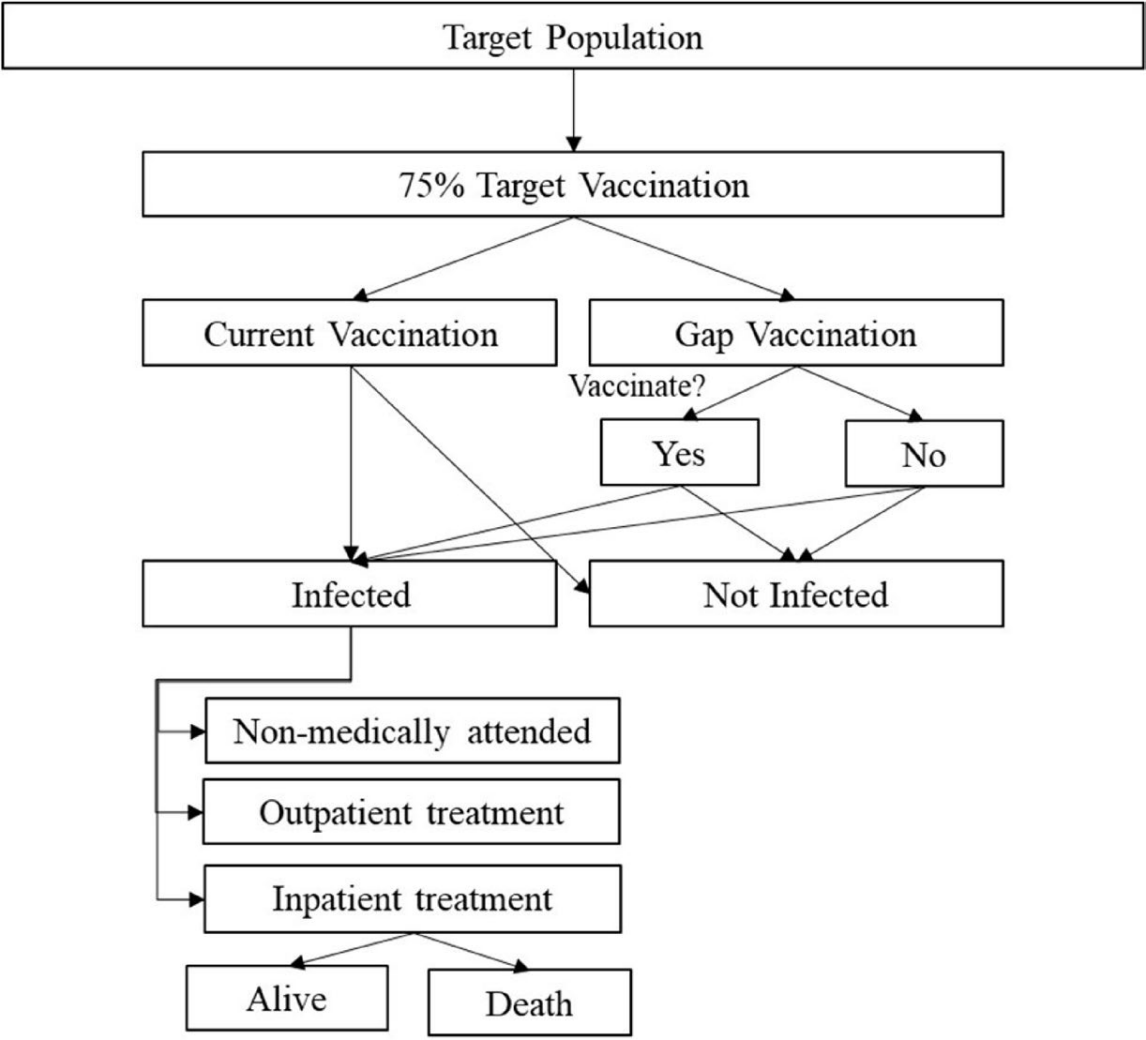
Decision flow of the epidemiological model

A static epidemiological model was developed to capture the clinical and economic consequences of seasonal influenza illness for WHO risk groups. The epidemiological model was constructed as a deterministic disease transition model in Microsoft Excel 365 MSO^©^ (Fig. 1). To denote the value of vaccination versus no vaccination, transition between states occur with different probabilities related to the reduced risk of influenza and its potential consequences. Algebraic computations display the potential benefits of achieving a 75% VCR, with an exhaustive number of details according to the country setting, subpopulation, and outcome of interest.

Starting with comprehensive epidemiological inputs from these risk groups, the model computed the number of individuals that would avoid influenza disease and its associated events (GP visits, hospitalisations, death and work absenteeism) as a result of achieving a particular VCR. The underlying direct medical and absenteeism costs from averted cases are presented at a country level and then combined. Disaggregated computations separately informed several subgroups of analysis, as follows:

- five WHO risk groups, as per the latest WHO Strategic Advisory Group of Experts recommendations for seasonal influenza vaccination [19]: older adults (aged ≥ 65 years), individuals with chronic conditions (e.g. HIV or AIDS, asthma, chronic heart or lung diseases), pregnant women, children aged 6–24 months and HCW.
- two alternative VCRs: the 2021/2022 VCR achieved in each country and the WHO-recommended 75% VCR target.
- four European countries: France, Italy, Spain, and the UK.

To avoid double counting individuals with chronic conditions, the populations sizes of pregnant women and HCWs were adjusted by excluding those with chronic conditions, as detailed in Clark et al. [20].

Model endpoints refer to averted: influenza cases; GP visits (any laboratory-confirmed consultations, or influenza-like illness consultations, adjusted with a positivity rate); hospitalisations (any hospitalisations coded as influenza or associated with influenza cardio-respiratory complications); case fatalities (based on excess death modelling associated with influenza); the underlying direct medical costs (relating to GP visits and hospitalisations); and the number of days of work lost and associated absenteeism-related costs.

This study builds upon the design from the publication by Preaud et al., which focused on 27 EU member states [17]. However, the current study has a reduced scope to four European countries, France, Italy, Spain and the UK, which represent nearly 50% of the 2022 EU and UK populations compared with Preaud et al. All inputs were revised and updated with recent local data for each risk group, where available.

### Data collection

Model inputs in four data clusters (population size, VCR data, epidemiological rates, and cost inputs; see Supplementary Material) were gathered using a dual approach based on a systematic literature review (SLR) and an additional manual search of local influenza surveillance systems and VCR monitoring schemes. The literature reviews were performed primarily to identify the epidemiological inputs and unitary cost rates. Key search terms were tested through Emtree searches to ensure relevance. Several equations were tested for each category of data and consistency checks were carried out by comparing results and relevant publications gathered previously through a manual search. Overall, two searches were conducted, one for each of the selected outcomes – clinical burden of the disease and economic burden of the disease. Searches and associated results are listed in Additional file 1: Table S1.1; Table S1.2. For the VCR data, multiple sources provided estimates per risk group, such as national public health agencies, pan-European surveys, as well as clinical and behavioural studies that collected patients’ immunisation status. A targeted literature review was performed to review the national public health agencies, as well as relevant European sources (Additional file 1: Supplementary Materials). Given the large range of model inputs required, a tailored approach was necessary to hierarchise the most relevant data from the most robust sources (Additional file 1: Supplementary Materials).

### Population characteristics

Studies were excluded if any of the following applied: unsuitable publication type (e.g. research group reports; white papers; book chapters; conference proceedings; thesis/dissertations; ongoing research; press articles [Additional file 1: Table S1.3]); reported regionally (except economic and VCR searches); included other countries or combined countries; years of data collection pre-dated the 2011–2012 season; or reported over the 2019–2020 season (to avoid inaccurate or misleading results due to the COVID-19 pandemic); or if they reported weekly or monthly. The full list of eligibility criteria is listed in Additional file 1: Table S1.3. The breakdown of at-risk populations by target group and country is available in the Supplementary Material (Additional file 1: Tables S2.1 and 2.2; Fig. S2.1).

### Model inputs

Detailed data collection methods and model inputs for the population-size inputs, VCR inputs, epidemiological inputs, vaccine efficacy and cost inputs are presented in the Supplementary Material (Additional file 1: Table S2.3).

For the VCR inputs, the latest available country-specific VCR data for the 2021/2022 seasonal influenza season were collected for each WHO risk group. For the epidemiological inputs, a 2018 meta-analysis from Somes et al. was selected as the source for influenza attack rates [4].

Values used as model inputs for vaccine efficacy are shown in Table 1. As the standard-dose (SD) quadrivalent influenza vaccine (QIV) is used as a standard of care for influenza vaccination across most of Europe, baseline figure vaccine efficacy for QIV-SD was derived from published SD trivalent (TIV) influenza vaccine efficacy values [21–23] (Table 1 and Additional file 1: Table S2.16). The use of vaccine efficacy estimates from meta-analysis of randomised controlled trials in a Cochrane review was preferred, in order to base the input on the highest level of evidence. This input was critical in the model and was tested in the deterministic sensitivity analysis (DSA). For a scenario analysis, high-dose (HD) relative vaccine efficacy values compared with SD were used for older adults in countries where HD is approved (France, Italy and Spain). In this scenario, QIV-HD was selected over QIV-SD due to clinical data indicating the potential superiority of QIV-HD across all strains in terms of efficacy compared with QIV-SD in adults aged ≥ 65 years [24–26] and the increasing availability of QIV-HD vaccines in the near future [16, 27]. Reductions in vaccine-induced immune responses in this population highlight the benefits of HD vaccinations [28]. For this later analysis, a relative vaccine efficacy of 24% for QIV-HD versus QIV-SD, as reported in a head-to-head randomised clinical trial, was applied [24].

**Table 1:** Baseline vaccine efficacy of the QIV-SD, by age group

Vaccination age group	Older adults (≥ 65 years)	6 months–17 years	18–65 years
Baseline vaccine efficacy, % (range)	60 (39–95)	67 (51–89)	61 (54–71)

*QIV-SD* Quadrivalent inactivated vaccine standard dose; *TIV* Trivalent inactivated vaccine

Source: Transformed from TIV vaccine efficacy (Jefferson et al. [21], Demicheli et al. [22], Demicheli et al. [23]) accounting for B-strain circulation, risk of mismatch, and cross-protection. Further details are available in the Supplementary Material

### Scenario and deterministic sensitivity analysis

Given the potential variability and uncertainty of particular inputs, DSA assessed the impact of all key variables on model outcomes (Table S4.1).

To further analyse the public health benefits and economic impact of achieving the 75% VCR for risk groups across France, Italy, Spain and the UK, a DSA was performed investigating univariate changes in the most sensitive parameters of the model (Additional file 1: Supplementary Materials). GP visit, hospitalisation and absenteeism rates were analysed with a range of ± 20% based on differences across seasons. Similarly, costs associated with GP visits and hospitalisations were analysed with a ± 20% range (± 20% arbitrary range, + 20% to show the increase in costs for an individual with chronic conditions).

**Fig. 2:**
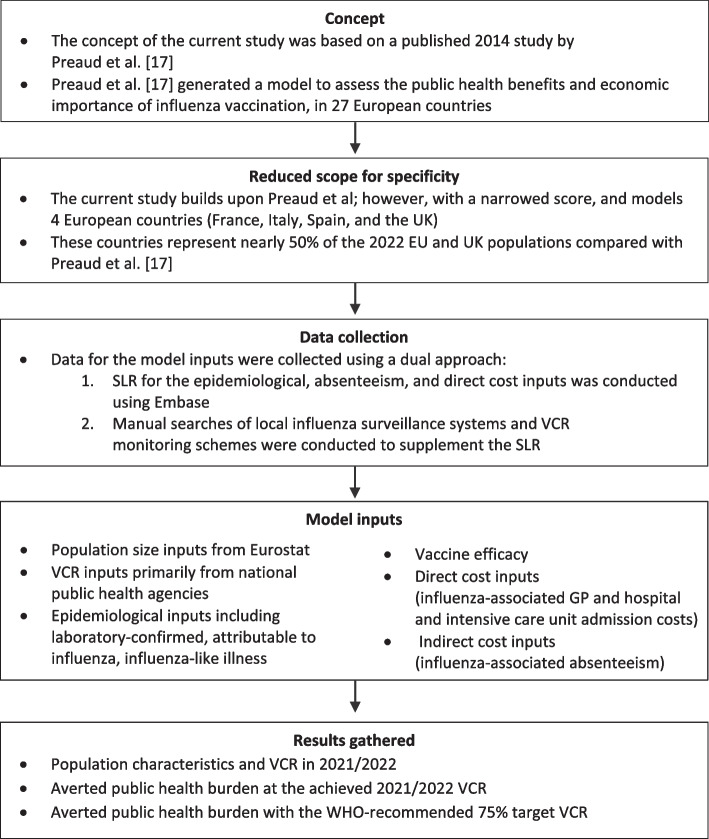
Summary of methodology. *EU* European; *GP* general practitioner; *SLR* systematic literature review; *UK* United Kingdom; *VCR* vaccine coverage rate

Within the scenario analysis, influenza-associated hospital admission rates were compared with those for excess influenza-associated hospitalisations for the older adult population as influenza can trigger cardio-respiratory complications, which can result in prolonged hospitalisations, medical support and eventually death. Hospital admission rates for influenza only were obtained from national public health reports, while excess influenza-associated hospitalisation rates were the same as per the main model epidemiological inputs. A summary of the methodological steps conducted in the current study are provided in Fig. 2

For illustrative purposes, an algebraic estimation of the potential investment needed to achieve 75% VCR in the at-risk groups was conducted by multiplying published average acquisition prices of vaccinations (QIV-SD) by the gap population between current VCR and 75% VCR.

## Results

### Population characteristics and VCR in 2021/2022

Two searches were completed (between January 2021 and March 2022), retrieving 5,508 hits for clinical burden of disease and 436 for economic burden from 2012 to 2020 (Additional file 1: Table S1.1). The total number of studies used for data extraction was 44 for clinical burden, and 37 for economic burden (Additional file 1: Fig. S1.1). Across the four countries, risk groups for seasonal influenza vaccination in the 2021/2022 season represented approximately 96 million individuals (Additional file 1: Table S2.2). Older adults (approximately 50 million individuals) accounted for 52% of this eligible population (53% in France, 57% in Italy, 51% in Spain and 48% in the UK), while individuals with chronic conditions (approximately 35.5 million individuals) accounted for 37% (34%, 35%, 40% and 40% in France, Italy, Spain and the UK, respectively) (Additional file 1: Table S2.2). Pregnant women (approximately 2 million individuals), children aged 6–24 months (approximately 3 million individuals) and HCW (approximately 5.5 million individuals) accounted for 2%, 3%, and 6% of the total eligible population, respectively.

The weighted average of influenza VCR across all risk groups was 47%, with substantial variation observed between countries (Additional file 1: Table S2.3). As of the 2021/2022 season, the UK achieved the highest weighted VCR across risk groups (65%), followed by Spain (47%), and then France and Italy (both 44%). Older adults had the highest VCR among eligible groups, with a weighted average of 66%; 82% in the UK, 69% in Spain, 58% in Italy and 57% in France. The highest VCR for children aged 6–24 months was observed in Italy (7.0%), compared with 6.8%, 4.9%, and 0.4% in Spain, France, and the UK, respectively.

**Fig. 3:**
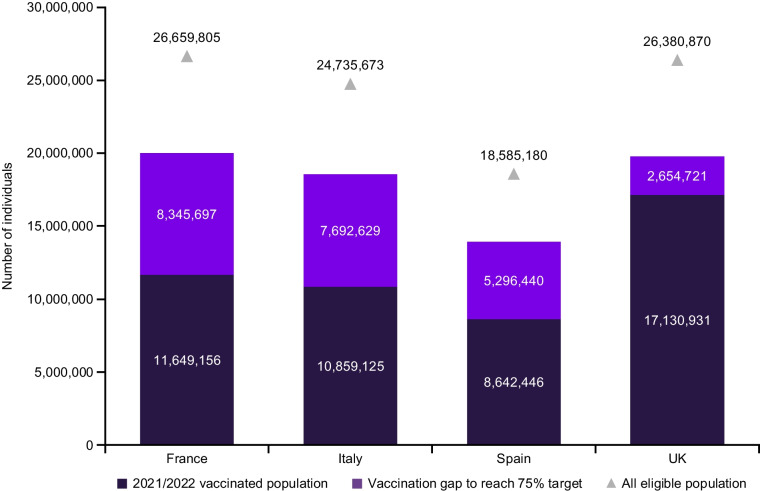
Vaccination gap to reach WHO-recommended 75% VCR target among all the eligible population for influenza vaccination. *UK* United Kingdom; *VCR* Vaccination coverage rate; *WHO* World Health Organization

When aggregating the VCR data for each risk-group across all four countries, 48.3 million people were estimated to receive influenza vaccination per year (Fig. 3). Thus, it was estimated that approximately an additional 24 million individuals would need to be vaccinated to achieve 75% VCR across all risk groups. Older adults and individuals with chronic conditions represented most of the unvaccinated population, accounting for 23% and 59% of the total, respectively. Pregnant women, children aged 6–24 months, and HCW accounted for 4%, 9%, and 8% of the unvaccinated population, respectively.

### Averted public health burden at the achieved 2021/2022 VCR

The 2021/2022 VCR was estimated to have averted approximately 1.9 million influenza cases, 375,200 GP visits, 73,200 hospital admissions and 38,400 deaths annually (Table 2). Based on age-specific influenza attack rates and vaccine efficacy values, 77% of influenza cases averted were in older adults, 19% in individuals with chronic conditions, 1% in pregnant women, 1% in children aged 6–24 months, and 3% in HCW (Table 2). Influenza cases averted at the 2021/2022 VCR are reported by risk group for each country in Additional file 1: Table S3.1. In Spain and Italy, older adults represented > 80% of the cases averted, accounting for 83% of all cases averted in Spain, and 82% in Italy respectively (Additional file 1: Table S3.1). In France, 77% of cases averted were in the older adult age group, and 69% of cases in the UK (Additional file 1: Table S3.1).

**Table 2:** Total number of influenza-related events and costs averted at the 2021/2022 VCR, by risk group and country

Influenza-related events averted	Older adults (≥ 65 years)	Individuals with chronic conditions	Pregnant women	Children (6–24 months)	HCW	France	Italy	Spain	UK	Total
Number of cases (%)	1,433,100 (77)	359,200 (19)	12,000 (1)	11,300 (1)	56,200 (3)	454,300 (24)	430,900 (23)	344,900 (18)	641,700 (34)	1,871,800
Number of GP visits (%)	188,000 (50)	159,800 (43)	4,000 (1)	3,300 (1)	20,100 (5)	168,500 (45)	45,200 (12)	89,000 (24)	72,500 (19)	375,200
Number of hospitalisations (%)	65,200 (89)	7,300 (10)	100 (0)	0 (0)	600 (1)	14,900 (20)	8,600 (12)	23,700 (32)	26,100 (36)	73,200
Number of deaths (%)	37,400 (97)	1,000 (3)	0 (0)	0 (0)	0 (0)	6,200 (16)	8,300 (22)	9,200 (24)	14,700 (38)	38,400
Number of days of work lost (%)	0 (0)	445,500 (82)	15,500 (3)	1,800 (0)	82,200 (15)	262,100 (48)	52,700 (10)	101,300 (19)	128,800 (24)	544,900
GP visit costs (%)	€8,382,200 (53)	€6,023,800 (38)	€237,000 (1)	€142,000 (1)	€1,029,400 (7)	€4,219,900 (27)	€1,046,400 (7)	€6,149,900 (39)	€4,398,200 (28)	€15,814,400
Hospitalisation costs (%)	€293,932,700 (92)	€22,619,600 (7)	€221,300 (0)	€63,600 (0)	€1,889,800 (1)	€86,478,300 (27)	€31,967,800 (10)	€94,813,600 (30)	€105,467,300 (33)	€318,727,000
Absenteeism costs (%)	€0 (0)	€50,722,300 (83)	€1,539,500 (3)	€295,300 (0)	€8,586,400 (14)	€30,676,400 (50)	€4,671,100 (8)	€8,344,500 (14)	€17,451,500 (29)	€61,143,500
All influenza-related costs (%)	€302,314,900 (76)	€79,365,700 (20)	€1,997,800 (1)	€500,900 (0)	€11,505,600 (3)	€121,374,600 (31)	€37,685,300 (10)	€109,308,000 (28)	€127,317,000 (32)	€395,684,900

*GP* General practitioner; *HCW* Healthcare worker; *UK* United Kingdom; *VCR* Vaccination coverage rate. Values in the table were rounded to the nearest hundred

Older adults and individuals with chronic conditions accounted for 50% and 43% of the GP visits averted with the 2021/2022 VCR, respectively (Table 2). Spain had the highest number of GP visits avoided for older adults, accounting for 61% of GP visits in that country, and France had the highest number of GP visits for individuals with chronic conditions, accounting for 52% of visits. Older adults accounted for 89% of the 73,200 estimated hospitalisations averted and 97% of the 38,400 estimated avoided deaths.

On average, each influenza GP visit costs €45 and influenza hospitalisation costs averaged at €3,651 per visit across the countries covered. Savings in direct costs achieved through the 2021/2022 VCR were estimated at €16 million for GP visits and €319 million for hospitalisations (Table 2), with estimated indirect cost savings of €61 million. Public health costs averted were greatest in the UK (€127 million), followed by France (€121 million), Spain (€109 million) and Italy (€38 million) (Table 2).

Older adults alone accounted for 90% of the direct cost savings (GP visits and hospitalisations) and 76% of the total averted costs, while individuals with chronic conditions accounted for 9% and 20% of the direct cost savings and total averted costs, respectively. Among individuals with chronic conditions, the subgroup of adults aged 50–64 years old accounted for 62% of the total costs saved for this risk group.

### Averted public health burden with the WHO-recommended 75% target VCR

Increasing the VCR to 75% from the 2020‒2021 VCR of each county (Additional file 1: Table S2.3) was estimated to avert an additional 918,200 cases of influenza each year. The greatest benefit would be observed in France (34% of averted cases) followed by Italy (30%), Spain (20%) and the UK (16%). Annually, an estimated 332,000 additional GP visits, 16,300 hospitalisations and 6,300 deaths could be averted (Table 3). Older adults represented 52% of the incremental avoidable hospitalisations and 80% of the incremental avoidable deaths.

**Table 3:** Total number of additional averted influenza-related events and costs by achieving the WHO 75% VCR target

Influenza-related events averted	Older adults (≥ 65 years)	Individuals with chronic conditions	Pregnant women	Children (6–24 months)	HCW	France	Italy	Spain	UK	Total
Number of cases (%)	237,500 (26)	408,800 (44)	26,200 (3)	193,200 (21)	52,500 (6)	316,700 (34)	274,900 (30)	180,300 (20)	146,300 (16)	918,200
Number of GP visits (%)	34,500 (10)	222,900 (67)	9,400 (3)	43,400 (13)	21,800 (7)	172,200 (52)	48,100 (15)	93,500 (28)	18,200 (5)	332,000
Number of hospitalisations (%)	8,500 (52)	6,700 (41)	100 (1)	700 (4)	300 (2)	6,150 (38)	3,350 (21)	4,200 (25)	2,600 (16)	16,300
Number of deaths (%)	5,000 (79)	1,300 (21)	0 (0)	20 (0)	0 (0)	2,200 (35)	2,700 (43)	1,200 (19)	200 (3)	6,300
Number of days of work lost (%)	0 (0)	586,500 (80)	35,900 (5)	24,800 (3)	87,600 (12)	375,900 (51)	98,600 (13)	204,600 (28)	55,700 (8)	734,800
GP visit costs (%)	€1,042,800 (8)	€9,236,400 (71)	€300,100 (2)	€1,856,600 (14)	€665,400 (5)	€4,423,200 (34)	€1,114,200 (9)	€6,458,900 (49)	€1,105,100 (8)	€13,101,300
Hospitalisation costs (%)	€41,986,300 (59)	€26,212,600 (37)	€282,900 (0)	€1,857,700 (3)	€1,028,600 (1)	€32,777,000 (46)	€12,546,250 (18)	€19,519,900 (27)	€6,525,100 (9)	€71,368,100
Absenteeism costs (%)	€0 (0)	€60,708,900 (77)	€3,966,900 (5)	€4,446,300 (6)	€9,571,300 (12)	€44,868,800 (57)	€8,829,200 (11)	€17,145,100 (22)	€7,850,400 (10)	€78,693,400
All influenza-related costs (%)	€43,029,100 (26)	€96,157,900 (59)	€4,549,900 (3)	€8,160,600 (5)	€11,265,300 (7)	€82,069,000 (50)	€22,489,500 (14)	€43,123,800 (26)	€15,480,500 (10)	€163,162,800

*GP* General practitioner; *HCW* Healthcare worker; *UK* United Kingdom; *VCR* Vaccination coverage rate; *WHO* World Health Organization. Values in the table were rounded to the nearest hundred

The associated economic impact was estimated as an additional €13 million saved for GP visits, €71 million saved for hospitalisations and €79 million saved for indirect costs. In total, the economic impact of achieving the 75% VCR target would represent an additional €163 million offset for influenza-related costs in the risk groups studied (Table 3). Most public health cost benefits would be delivered in France, with €82 million in total savings, followed by Spain with €43 million, Italy with €22 million and the UK with €15 million (Table 3). Overall, older adults and individuals with chronic conditions accounted for the largest proportion of the avoidable economic burden of influenza, accounting for 51% and 42% of the direct costs and 26% and 59% of total costs saved, respectively.

### Scenario and deterministic sensitivity analysis

In a scenario in which vaccination rates are improved from the 2021/2022 rate to the 75% VCR (i.e., in which the ‘gap’ between the two is bridged) and QIV-HD is used instead of QIV-SD alone in older adults, an estimated 975,000 influenza cases, 340,000 GP visits, 18,000 hospital admissions and 7,500 deaths would be averted. This change from QIV-SD to QIV-HD translates into savings of €95 million for direct medical costs and €79 million for absenteeism costs (Additional file 1: Table S5.1).

**Fig. 4:**
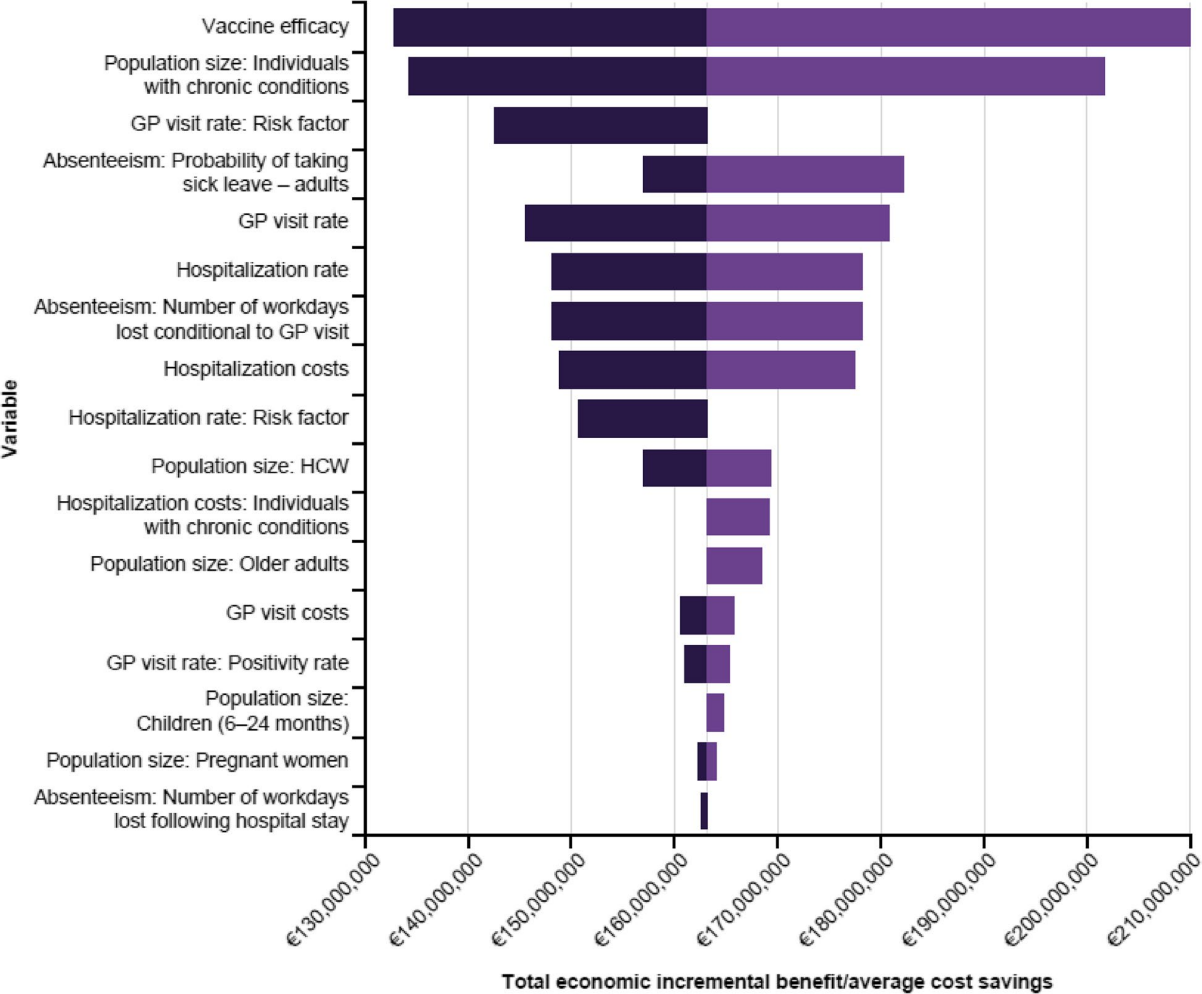
DSA of the average incremental costs or cost savings of increasing the VCR to 75%. *DSA* Deterministic sensitivity analysis; *GP* General practitioner; *HCW* Healthcare worker; *VCR* Vaccination coverage rate

Using the DSA to assess total economic incremental benefit/average cost savings (lower boundary; upper boundary), vaccine efficacy (€133 million–€210 million) was the main variable contributing to savings, followed by the population size of individuals with chronic conditions (€134 million–€202 million) and GP visit rate (€146 million–€181 million) (Fig. 4).

The comparison between influenza-associated hospital administration rates and excess influenza-associated hospitalisations demonstrated that hospitalisations due to influenza complications avoided at the 2021/2022 VCR are three times higher than influenza-only hospitalisations (Additional file 1: Fig. S5.1).

The estimation of the potential investment needed to achieve 75% VCR in at-risk groups is reported in Additional file 1: Table S6.1. However, as the value of vaccination is best estimated in terms of long-term costs and quality-adjusted life years in the context of a willingness-to-pay threshold, these illustrative estimates should be interpreted cautiously.

## Discussion

Modelling the impact of influenza vaccination, particularly in high-risk groups, is important to support vaccine implementation and inform resource allocation. This epidemiological model based on the 2021/2022 VCR, showed that approximately 1.9 million influenza cases, 73,200 hospitalisations and 38,400 deaths were avoided across France, Italy, Spain and the UK. However, the weighted average 2021/2022 VCR (47%) was much lower than the WHO target of 75%. Increasing the seasonal influenza VCR to this 75% target in all WHO-recommended risk groups would achieve substantially greater public health and economic benefits in these countries, due to the reduction in influenza disease burden in risk groups and in the cost of annual influenza epidemics (due to reductions in lost productivity and absenteeism) [17]. As part of its Global Influenza Strategy 2019–2030, the WHO aims to continue supporting countries to implement national immunisation policies for risk groups, as recommended by the Strategic Advisory Group of Experts on Immunization, and to monitor uptake through national databases [29].

Older adults represented the greatest clinical and economic burden in terms of influenza cases, hospitalisations and deaths. Given local vaccine recommendations and the contribution of older adults to the total estimated clinical and economic outcomes, an analysis scenario applying QIV-HD in older adults was considered relevant. The efficacy of QIV-HD relative to QIV-SD was 24%, in the prevention of laboratory-confirmed cases of influenza or influenza-like illness, in older adults (aged ≥ 65 years old) [23]. This suggests that one quarter of all breakthrough influenza could be prevented if QIV-HD was used over QIV-SD, and evidences the potential alleviation of economic burden within the population. Previous studies have indicated that using QIV-HD over QIV-SD may be cost-effective in several European settings for this at-risk population [30, 31], and may contribute to additional public health and economic benefits beyond those calculated in this study, such as reduced rates of influenza-related secondary bacterial infections, functional decline and poor pregnancy outcomes, and improved child education and macroeconomic stability due to reduced absenteeism [32].

Given the increasingly ageing population (population aged ≥ 65 years is projected to increase by 12% between 2022 and 2030) [33], a growing prevalence of chronic underlying conditions and emerging respiratory virus threats, the need to protect vulnerable adults across Europe is imperative [34]. Given these trends, modelled estimates in this study may represent only a fraction of the future influenza-related burden and costs of disease.

Compared with the previous findings [17], our model found an increased public health burden, which may be possible to avert by achieving the WHO target of 75% VCR. Achieving a 75% VCR in the four selected countries led to 54% fewer influenza cases (compared with achieving the 75% VCR in the Preaud et al. model from 2014 [17]), 5% more GP visits, 134% fewer hospital admissions and 90% fewer deaths. Prevention of these additional influenza cases and events through achieving the 75% VCR also translates into 205% and 29% lower direct and indirect cost savings, respectively, compared with the previous model [17]. These differences may be explained by the higher proportion of older adults in the current population versus the previous study population, use of epidemiological excess rates, a higher vaccine efficacy for QIV-SD versus trivalent inactivated vaccine standard dose (TIV-SD), and fewer GP visits.

In this study population, older adults represented a large proportion of the influenza cases, hospitalisations, GP visits, and nearly all of the deaths avoided at the 2020/2021 VCR or by achieving a 75% VCR. Older adults and individuals with chronic conditions accounted for the largest proportion of the avoidable economic burden of influenza. Therefore, it is imperative that HCWs prioritise vaccination of these subgroups, to maximise the public health impact and reduce the economic burden [35].

Influenza is a leading cause of work absenteeism, yet is frequently overlooked by conventional surveillance systems, which rely on healthcare data from GP or hospital records [36, 37]. Capturing data on individuals who do not seek medical attention will therefore enhance influenza reporting [36, 37]. Uhart et al. modelled the distribution of cost savings from a societal perspective if the QIV-SD vaccine was used instead of TIV-SD across Europe [38]. In Europe, the VCR in working adults remains far lower than the VCR seen among older adults in a context where, unlike in the United States (US) and Canada, there is no universal influenza vaccination reimbursement [39].

This study utilised an influenza VCR considered to be reflective of a post-COVID-19 scenario. COVID-19 vaccination and surveillance provided an opportunity to improve other adult immunisation programmes, reinforce infrastructures and assess potential synergies between COVID-19 and influenza management strategies, including enhanced epidemiological surveillance [19]. Well-established adult influenza vaccination programmes proved to be a key component of the success of the response to the COVID-19 pandemic by facilitating access to and acceptance of mass vaccination campaigns [40], highlighting that implementing annual adult immunisation programmes could be mutually beneficial in protecting vulnerable adults against a variety of respiratory pathogens, including influenza, severe acute respiratory syndrome coronavirus 2 (SARS CoV-2), pertussis, pneumococcal diseases and respiratory syncytial viruses. As suggested by the Board of the Vaccination Calendar for Life in Italy, an innovative and concerted model based on co-administration of adult vaccines should ensure immunisation reaches vulnerable populations, in social and health residential facilities, and at home [41].

Following the COVID-19 pandemic and vaccine rollouts, lessons can be learned in terms of how to drive vaccine uptake, particularly for vulnerable populations [42]. Countries such as the UK, Portugal and Spain achieved record influenza VCRs during the 2020/2021 and 2021/2022 influenza seasons, thus increasing the benefits of influenza prevention at a time when healthcare systems were particularly under stress [43]. Furthermore, the experience of the pandemic has highlighted the importance of identification of risk groups, namely, people more at risk of experiencing complications from infectious diseases, therefore warranting increased vaccination efforts, and reinforcing the importance of high adult vaccination coverage as a tool for pandemic preparedness. The US Biomedical Advanced Research and Development Authority has recently set goals of accelerating vaccine development and production, as well as improving vaccine performance [44, 45]. Newly introduced influenza vaccines have been shown to provide better protection for vulnerable populations with HD vaccines showing increased benefits in older adults [25]. In parallel, the medical community is looking towards a future research and development roadmap for novel influenza vaccines, which it expects, among other improvements, to lead to better protection and reduced production times [46].

Several limitations apply to our analysis. Due to its static nature, our model does not account for the impact of vaccination on the reduction of the force of infection (i.e., the rate at which susceptible individuals in population acquire an infection disease), also called the indirect effect of vaccination, benefiting primarily the unvaccinated population. Hence, our result may be considered as an underestimation of the true potential impact of influenza vaccination. Also, as QIV-SD efficacy data were not uniformly available for all selected risk groups, a proxy based on TIV-SD efficacy in randomised trials (estimated by meta-analysis in Cochrane reviews) had to be used, adjusting for the benefit of protection against both B lineages [47]. In addition, due to data paucity, several of the influenza VCRs and epidemiological and cost inputs that were used may not precisely match the risk group, period, and country considered; in those cases, a potential underestimation of the real burden can exist, as the study prioritised conservative assumptions. Influenza is also a significant driver of emergency visits and intensive care admissions, but available data (from surveillance systems and literature) does not allow for accurate evaluation of the overall impact on healthcare systems and the proportion of these events potentially avoided by vaccination [48]. When combined with COVID-19, respiratory syncytial virus and other pathogens, influenza exerts a compounded pressure during winter and contributes to the overall saturation and disruption of healthcare systems, another aspect that was not modelled in this study [49, 50]. Lastly, the estimated cost of vaccine acquisition provided in this study should be noted as a limitation. The cost of vaccine acquisition is a single component of the resources necessary for vaccine implementation, with additional resources required for vaccine application and immunisation campaigns. Due to the complexities associated with obtaining the necessary local data to provide accurate estimates, the estimated costs are unlikely to reflect the real value of vaccine acquisition for payors and could be easily misinterpreted in the context of this research.

## Conclusions

Across France, Italy, Spain and the UK, the seasonal influenza VCR remains below the 75% target recommended by the WHO, with substantial heterogeneity across countries and risk groups [17, 51, 52]. Despite suboptimal coverage, vaccination had a considerable positive impact on reducing overall influenza-related burden, resulting in cost savings.

By achieving the recommended 75% VCR, twice as many influenza cases could be prevented, avoiding thousands of hospitalisations and physician visits, and thereby reducing the burden on healthcare systems. Importantly, this study revealed that older adults account for the majority of preventable cases and deaths, along with those with chronic conditions, highlighting the need for health authorities and HCWs to prioritise these populations during their efforts to increase influenza vaccination uptake. By doing so, the public health and economic burdens associated with influenza could be substantially reduced. With an ageing population, pressured healthcare systems and budget constraints, the economic benefits of reducing influenza cases and the associated complications are of paramount importance.

### Supplementary Information

**Supplementary material 1.**

## Data Availability

The authors can confirm that all data sources, model inputs and results are included in the article and its supplementary information files.

## Abbreviations

DSA: Deterministic sensitivity analysis
EU: European Union
GP: General practitioner
HCW: Healthcare worker
HD: High dose
QIV-HD: Quadrivalent inactivated vaccine high dose
QIV-SD: Quadrivalent inactivated vaccine standard dose
SLR: Systematic literature review
TIV-SD: Trivalent inactivated vaccine standard dose
US: United States
VCR: Vaccination coverage rate
WHO: World Health Organization

## References

[1] World Health Organization. Influenza. https://www.who.int/teams/health-product-policy-and-standards/standards-and-specifications/vaccines-quality/influenza#:~:text=Both%20influenza%20A%20and%20B,20%2D30%25%20in%20children. Accessed 30 Jan 2024.

[2] World Health Organization. Influenza (seasonal). 2023. https://www.who.int/news-room/fact-sheets/detail/influenza-(seasonal). Accessed 30 Jan 2024.

[3] Iuliano AD, Roguski KM, Chang HH, Muscatello DJ, Palekar R, Tempia S (2018). Estimates of global seasonal influenza-associated respiratory mortality: a modelling study. Lancet.

[4] Somes MP, Turner RM, Dwyer LJ, Newall AT (2018). Estimating the annual attack rate of seasonal influenza among unvaccinated individuals: a systematic review and meta-analysis. Vaccine.

[5] World Health Organization (2012). Vaccines against influenza WHO position paper — November 2012. Wkly Epidemiol Rec.

[6] Kuster SP (2011). Incidence of influenza in healthy adults and healthcare workers: a systematic review and meta-analysis. PLoS ONE.

[7] Coleman BL, Fadel SA, Fitzpatrick T, Thomas SM (2018). Risk factors for serious outcomes associated with influenza illness in high- versus low- and middle-income countries: systematic literature review and meta-analysis. Influenza Other Respir Viruses.

[8] Fell DB, Azziz-Baumgartner E, Baker MG, Batra M, Beauté J, Beutels P (2017). Influenza epidemiology and immunization during pregnancy: final report of a World Health Organization working group. Vaccine.

[9] Near AM, Tse J, Young-Xu Y, Hong DK, Reyes CM (2022). Burden of influenza hospitalization among high-risk groups in the United States. BMC Health Serv Res.

[10] Lina B, Georges A, Burtseva E, Nunes MC, Andrew MK, McNeil SA (2020). Complicated hospitalization due to influenza: results from the Global Hospital Influenza Network for the 2017–2018 season. BMC Infect Dis.

[11] Lemaitre M, Fouad F, Carrat F, Crépey P, Gaillat J, Gavazzi G (2022). Estimating the burden of influenza-related and associated hospitalizations and deaths in France: an eight-season data study, 2010–2018. Influenza Other Respir Viruses.

[12] Froes F, Carmo M, Lopes H, Bizouard G, Gomes C, Martins M (2022). Excess hospitalizations and mortality associated with seasonal influenza in Portugal, 2008–2018. BMC Infect Dis.

[13] Ryan J, Zoellner Y, Gradl B, Palache B, Medema J (2006). Establishing the health and economic impact of influenza vaccination within the European Union 25 countries. Vaccine.

[14] World Health Organization. Managing seasonal vaccination policies and coverage in the European Region. 2023. https://www.who.int/europe/activities/managing-seasonal-vaccination-policies-and-coverage-in-the-european-region. Accessed 18 Dec 2023.

[15] The Council of the European Union. Council recommendation of 22 December 2009 on seasonal influenza vaccination. 2009. https://eur-lex.europa.eu/LexUriServ/LexUriServ.do?uri=OJ:L:2009:348:0071:0072:EN:PDF#:~:text=Member%20States%20are%20encouraged%20to,coverage%20rate%20of%2075%20%25%20for%20'. Accessed 30 Jan 2024.

[16] European Centre for Disease Prevention and Control. Seasonal influenza vaccination recommendations and coverage rates in EU/EEA Member States. 2023. https://www.ecdc.europa.eu/en/publications-data/seasonal-influenza-vaccination-recommendations-and-coverage-rates-eueea-member. Accessed 30 Jan 2024.

[17] Preaud E, Durand L, Macabeo B, Farkas N, Sloesen B, Palache A (2014). Annual public health and economic benefits of seasonal influenza vaccination: a European estimate. BMC Public Health.

[18] Liu Y, Sandmann FG, Barnard RC, Pearson CAB, Pastore R, Pebody R, CMMID COVID-19 Working Group (2022). Optimising health and economic impacts of COVID-19 vaccine prioritisation strategies in the World Health Organization European Region: a mathematical modelling study. Lancet Reg Health Eur..

[19] World Health Organization (2020). WHO SAGE Seasonal influenza vaccination recommendations during the COVID-19 pandemic. Wkly Epidemiol Rec.

[20] Clark A, Jit M, Warren-Gash C, Guthrie B, Wang HH, Mercer S (2020). Global, regional, and national estimates of the population at increased risk of severe COVID-19 due to underlying health conditions in 2020: a modelling study. Lancet Glob Health.

[21] Jefferson T, Di Pietrantonj C, Al-Ansary LA, Ferroni E, Thorning S, Thomas RE (2010). Vaccines for preventing influenza in the elderly. Cochrane Database Syst Rev..

[22] Demicheli V, Jefferson T, Ferroni E, Rivetti A, Di Pietrantonj C (2018). Vaccines for preventing influenza in healthy adults. Cochrane Database Syst Rev..

[23] Demicheli V, Jefferson T, Di Pietrantonj C, Ferroni E, Thorning S, Thomas RE (2018). Vaccines for preventing influenza in the elderly. Cochrane Database Syst Rev..

[24] DiazGranados CA, Dunning AJ, Kimmel M, Kirby D, Treanor J, Collins A (2014). Efficacy of high-dose versus standard-dose influenza vaccine in older adults. N Engl J Med.

[25] European Centre for Disease Prevention and Control. Systematic review of the efficacy, effectiveness and safety of newer and enhanced seasonal influenza vaccines for the prevention of laboratory-confirmed influenza in individuals aged 18 years and over. https://www.ecdc.europa.eu/sites/default/files/documents/seasonal-influenza-vaccines-systematic-review-efficacy.pdf. Accessed 29 Jan 2024.

[26] Recommendations by the Standing Committee on Vaccination. Recommendations by the Standing Committee on Vaccination (STIKO) at the Robert Koch Institute – 2023. Epidemiologisches Bulletin. https://www.rki.de/EN/Content/infections/Vaccination/recommandations/04_23_englisch.pdf?__blob=publicationFile. Accessed 13 Dec 2023.

[27] de Boer P, van Maanen BM, Damm O, Ultsch B, Dolk FCK, Crépey P (2017). A systematic review of the health economic consequences of quadrivalent influenza vaccination. Expert Rev Pharmacoecon Outcomes Res.

[28] McElhaney JE, Verschoor CP, Andrew MK, Haynes L, Kuchel GA, Pawelec G (2020). The immune response to influenza in older humans: beyond immune senescence. Immun Ageing.

[29] World Health Organization. Global Influenza Strategy 2019–2030. 2019. https://apps.who.int/iris/bitstream/handle/10665/311184/9789241515320eng.pdf?sequence=18&isAllowed=y. Accessed 30 Jan 2024.

[30] Alvarez FP, Chevalier P, Borms M, Bricout H, Marques C, Soininen A (2023). Cost-effectiveness of influenza vaccination with a high dose quadrivalent vaccine of the elderly population in Belgium, Finland, and Portugal. J Med Econ.

[31] Rumi F, Basile M, Cicchetti A (2021). Cost-effectiveness and budget impact analysis for high dose quadrivalent influenza vaccine in the Italian elderly population. Glob Reg Health Technol Assess.

[32] Macias AE, McElhaney JE, Chaves SS, Nealon J, Nunes MC, Samson SI (2021). The disease burden of influenza beyond respiratory illness. Vaccine.

[33] Eurostat: Population total Eurostat. https://ec.europa.eu/eurostat/data/database. Accessed 25 Jan 2024.

[34] European Comission. Chronic diseases – The health challenge of our times. European Union. 2014. https://health.ec.europa.eu/publications/chronic-diseases_en. Accessed 30 Nov 2023.

[35] World Heath Organization. Influenza - COVID-19 interface. https://www.who.int/teams/global-influenza-programme/influenza-covid19. Accessed 25 Jan 2024.

[36] Groenewold MR, Konicki DL, Luckhaupt SE, Gomaa A, Koonin LM (2013). Exploring national surveillance for health-related workplace absenteeism: lessons learned from the 2009 influenza A pandemic. Disaster Med Public Health Prep.

[37] Groenewold MR. Using worker absenteeism to track the flu. 2019. https://blogs.cdc.gov/niosh-science-blog/2019/07/16/flu-absent/. Accessed 31 Oct 2023.

[38] Uhart M, Bricout H, Clay E, Largeron N (2016). Public health and economic impact of seasonal influenza vaccination with quadrivalent influenza vaccines compared to trivalent influenza vaccines in Europe. Hum Vaccin Immunother.

[39] Ortiz JR, Perut M, Dumolard L, Wijesinghe PR, Jorgensen P, Ropero AM (2016). A global review of national influenza immunization policies: analysis of the 2014 WHO/UNICEF Joint Reporting Form on immunization. Vaccine.

[40] Morales KF, Brown DW, Dumolard L, Steulet C, Vilajeliu A, Ropero Alvarez AM (2021). Seasonal influenza vaccination policies in the 194 WHO Member States: the evolution of global influenza pandemic preparedness and the challenge of sustaining equitable vaccine access. Vaccine X.

[41] Bonanni P, Angelillo IF, Villani A, Biasci P, Scotti S, Russo R (2021). Maintain and increase vaccination coverage in children, adolescents, adults and elderly people: let's avoid adding epidemics to the pandemic: appeal from the Board of the Vaccination Calendar for Life in Italy: maintain and increase coverage also by re-organizing vaccination services and reassuring the population. Vaccine.

[42] Kassianos G, Banerjee A, Baron-Papillon F, Hampson AW, McElhaney JE, McGeer A, et al. Key policy and programmatic factors to improve influenza vaccination rates based on the experience from four high-performing countries. Drugs Context. 2021;10:2020–9–5. 10.7573/dic.2020-9-5 PMC7789908 33456480

[43] Kong G, Lim NA, Chin YH, Ng YPM, Amin Z (2022). Effect of COVID-19 Pandemic on Influenza Vaccination Intention: A Meta-Analysis and Systematic Review. Vaccines.

[44] US Biomedical Advanced Research and Development Authority. BARDA Strategic Plan 2022–2026. HHS ASPR BARDA. 2022. https://www.medicalcountermeasures.gov/barda/strategic-plan/#plan. Accessed 30 Nov 2023.

[45] Newland M, Durham D, Asher J, Treanor JJ, Seals J, Donis RO (2021). Improving pandemic preparedness through better, faster influenza vaccines. Expert Rev Vaccines.

[46] Moore KA, Ostrowsky JT, Kraigsley AM, Mehr AJ, Bresee JS, Friede MH (2021). A Research and Development (R&D) roadmap for influenza vaccines: looking toward the future. Vaccine.

[47] Jefferson T, Di Pietrantonj C, Rivetti A, Bawazeer GA, Al-Ansary LA, Ferroni E (2010). Vaccines for preventing influenza in healthy adults. Cochrane Database Syst Rev..

[48] Rigoine de Fougerolles T, Damm O, Ansaldi F, Chironna M, Crépey P, de Lusignan S (2022). National influenza surveillance systems in five European countries: a qualitative comparative framework based on WHO guidance. BMC Public Health..

[49] Alosaimi B, Naeem A, Hamed ME, Alkadi HS, Alanazi T, Al Rehily SS (2021). Influenza co-infection associated with severity and mortality in COVID-19 patients. Virol J.

[50] Burki TK (2021). Circulation of influenza, RSV, and SARS-CoV-2: an uncertain season ahead. Lancet Respir Med.

[51] European Commission. Proposal for a Council recommendation on seasonal influenza vaccination 2009. https://op.europa.eu/en/publication-detail/-/publication/9947fdfdb9-ce99-4064-9711-44385d555c1b/language-en. Accessed 30 Jan 2024.

[52] Oakley S, Bouchet J, Costello P, Parker J (2021). Influenza vaccine uptake among at-risk adults (aged 16–64 years) in the UK: a retrospective database analysis. BMC Public Health.

