# Supplementary material for: Public health and economic benefits of seasonal influenza vaccination in risk groups in France, Italy, Spain and the UK: state of play and perspectives
Source: BMC Public Health. 2024 May 3;24:1222. doi: 10.1186/s12889-024-18694-5 (PMC11067100; doi:10.1186/s12889-024-18694-5)
Supplement: Supplementary file 1 — Supplementary material 1. [file 12889_2024_18694_MOESM1_ESM.zip › Preaud et al Study update_Supplementary Materials_updated_18April24_clean.docx]

**Supplementary materials**

**Public health and economic benefits of seasonal influenza vaccination in risk groups in France, Italy, Spain, and the UK: State of play and perspectives**

Thierry Rigoine de Fougerolles^1^, Théophile Baïssas^2^, Guillaume Perquier^1^,
Olivier Vitoux^1,3^, Pascal Crépey^3^, José Bartelt-Hofer^4*^, Hélène Bricout^4^, and
Audrey Petitjean^4^

^1^Corporate Value Associates, Paris, France; ^2^Corporate Value Associates, London, United Kingdom; ^3^Univ Rennes, EHESP, CNRS, Inserm, Arènes - UMR 6051, RSMS – U1309, Rennes, France; ^4^Sanofi, Lyon, France.

*Corresponding author

# 1. Data collection and classification of evidence

A dual approach was implemented to collect data for vaccination coverage rate (VCR), epidemiological and direct cost inputs relying on a literature review on Embase and a complementary search of European and national influenza surveillance systems and VCR monitoring systems.

A systematic literature review for the epidemiological, absenteeism, and direct cost inputs was conducted using Embase. The relevant literature was identified by following a rigorous search protocol, designed to facilitate both auditing and reproducibility. While all corresponding search results were extracted, Embase filters were used to focus on eligible search results that were articles, reviews, and articles in press.

Scientific communications identified between the time the Embase literature review was performed (January 2021) and the time the model was finalised (March 2022) were included. The collection period included articles published in 2012–2020, with an analyst performing a first screening and a senior investigator conducting a full independent review to ensure suitability of the selected articles after each screening step. Scientific communications corresponding to posters presented at conferences in 2021 were referenced, despite meeting an exclusion criterion from the literature review.

Key search terms were tested through Emtree searches to ensure relevance. Several equations were tested for each category of data. Consistency checks were carried out by comparing results and relevant publications gathered previously through a manual search. The terms for each category were tested individually and collectively in combination with the term influenza, either present in all fields of the publication or restricted to the title and abstract.

Overall, two searches were conducted, one for each of the selected outcomes – clinical burden of the disease and economic burden of the disease. These were run using a full selection of the search terms, which were either Emtree terms or searched only in the title and abstract fields of relevant publications. The scope was extended to European Union (EU) 27 countries and the UK to ensure having datapoints for France, Italy, Spain and the UK, by filling the gaps with neighbouring countries if needed. Searches and associated results are listed in **Table S1.1**.

| Table S1.1 Data collection – Search equations and results for the two data searches conducted on Embase | | | | | | |
| --- | --- | --- | --- | --- | --- | --- |
| **Data cluster** | **Disease** | **Burden** | **Publication year** | **Territory** | **Total search results** | **Eligible search results** |
| Epidemiological input | Influenza:ti,ab | disease burden’[Emtree] OR 'epidemiology'[Emtree] OR 'mortality'[Emtree] OR 'morbidity'[Emtree] OR 'incidence'[Emtree] OR 'infection'[Emtree] OR 'consultation'[Emtree] OR 'hospital admission'[Emtree] OR 'hospitalisation'[Emtree] OR 'intensive care unit'[Emtree] OR 'flu like syndrome'[Emtree] OR 'respiratory tract infection'[Emtree] OR 'mortality '[Emtree] OR 'death’[Emtree] OR 'cases':ti,ab OR 'outbreak':ti,ab OR 'attack rate':ti,ab | 2012:py OR 2013:py OR 2014:py OR 2015:py OR 2016:py OR 2017:py OR 2018:py OR 2019:py OR 2020:py | global':ti OR 'meta-analysis':ti OR 'review':ti OR 'europe':ti,ab,kw OR 'european':ti,ab,kw OR 'france':ti,ab,kw OR 'french':ti,ab,kw OR 'germany':ti,ab,kw OR 'german':ti,ab,kw OR 'italy':ti,ab,kw OR 'italian':ti,ab,kw OR 'spain':ti,ab,kw OR 'spanish':ti,ab,kw OR 'united kingdom':ti,ab,kw OR 'england':ti,ab,kw OR 'english':ti,ab,kw OR 'belgium':ti,ab,kw OR 'belgian':ti,ab,kw OR 'poland':ti,ab,kw OR 'polish':ti,ab,kw OR 'romania':ti,ab,kw OR 'romanian':ti,ab,kw OR 'greece':ti,ab,kw OR 'greek':ti,ab,kw OR 'sweden':ti,ab,kw OR 'swedish':ti,ab,kw OR 'czech republic':ti,ab,kw OR 'czech':ti,ab,kw OR 'portugal':ti,ab,kw OR 'portuguese':ti,ab,kw OR 'croatia':ti,ab,kw OR 'croatian':ti,ab,kw OR 'bulgaria':ti,ab,kw OR 'bulgarian':ti,ab,kw OR 'hungary':ti,ab,kw OR 'hungarian':ti,ab,kw OR 'finland':ti,ab,kw OR 'finnish':ti,ab,kw OR 'austria':ti,ab,kw OR 'austrian':ti,ab,kw OR 'denmark':ti,ab,kw OR 'danish':ti,ab,kw OR 'ireland':ti,ab,kw OR 'irish':ti,ab,kw OR 'lithuania':ti,ab,kw OR 'lithuanian':ti,ab,kw OR 'latvia':ti,ab,kw OR 'latvian':ti,ab,kw OR 'slovakia':ti,ab,kw OR 'slovak':ti,ab,kw OR 'cyprus':ti,ab,kw OR 'cypriot':ti,ab,kw OR 'luxembourg':ti,ab,kw OR 'luxembourgers':ti,ab,kw OR 'malta':ti,ab,kw OR 'maltese':ti,ab,kw OR 'slovenia':ti,ab,kw OR 'slovene':ti,ab,kw OR 'estonia':ti,ab,kw OR 'estonian':ti,ab,kw OR 'netherlands':ti,ab,kw OR 'dutch':ti,ab,kw | 5,508 | 4,327 |
| Economic burden | Influenza:ti,ab | ‘health economics’[EmTree] OR ‘health care cost’ [EmTree] OR ‘cost’ [EmTree] OR ‘cost benefit analysis’ [EmTree] OR ‘absenteeism’ [EmTree] OR ‘presenteeism’ [EmTree] OR ‘medical leave’ [EmTree] OR ‘productivity’ [EmTree] OR ‘sick leave’:ti,ab 2012:py OR 2013:py OR 2014:py OR 2015:py OR 2016:py OR 2017:py OR 2018:py OR 2019:py OR 2020:py | 2012:py OR 2013:py OR 2014:py OR 2015:py OR 2016:py OR 2017:py OR 2018:py OR 2019:py OR 2020:py | europe':ti,ab,kw OR 'european':ti,ab,kw OR 'france':ti,ab,kw OR 'french':ti,ab,kw OR 'germany':ti,ab,kw OR 'german':ti,ab,kw OR 'italy':ti,ab,kw OR 'italian':ti,ab,kw OR 'spain':ti,ab,kw OR 'spanish':ti,ab,kw OR 'united kingdom':ti,ab,kw OR 'england':ti,ab,kw OR 'english':ti,ab,kw OR 'belgium':ti,ab,kw OR 'belgian':ti,ab,kw OR 'poland':ti,ab,kw OR 'polish':ti,ab,kw OR 'romania':ti,ab,kw OR 'romanian':ti,ab,kw OR 'greece':ti,ab,kw OR 'greek':ti,ab,kw OR 'sweden':ti,ab,kw OR 'swedish':ti,ab,kw OR 'czech republic':ti,ab,kw OR 'czech':ti,ab,kw OR 'portugal':ti,ab,kw OR 'portuguese':ti,ab,kw OR 'croatia':ti,ab,kw OR 'croatian':ti,ab,kw OR 'bulgaria':ti,ab,kw OR 'bulgarian':ti,ab,kw OR 'hungary':ti,ab,kw OR 'hungarian':ti,ab,kw OR 'finland':ti,ab,kw OR 'finnish':ti,ab,kw OR 'austria':ti,ab,kw OR 'austrian':ti,ab,kw OR 'denmark':ti,ab,kw OR 'danish':ti,ab,kw OR 'ireland':ti,ab,kw OR 'irish':ti,ab,kw OR 'lithuania':ti,ab,kw OR 'lithuanian':ti,ab,kw OR 'latvia':ti,ab,kw OR 'latvian':ti,ab,kw OR 'slovakia':ti,ab,kw OR 'slovak':ti,ab,kw OR 'cyprus':ti,ab,kw OR 'cypriot':ti,ab,kw OR 'luxembourg':ti,ab,kw OR 'luxembourgers':ti,ab,kw OR 'malta':ti,ab,kw OR 'maltese':ti,ab,kw OR 'slovenia':ti,ab,kw OR 'slovene':ti,ab,kw OR 'estonia':ti,ab,kw OR 'estonian':ti,ab,kw OR 'netherlands':ti,ab,kw OR 'dutch':ti,ab,kw | 436 | 323 |

A Preferred Reporting Items for Systematic Reviews and Meta-Analyses (PRISMA) diagram was created to illustrate the selection process **(Fig. S1.1**) For clinical burden and economic burden searches, results were filtered on Embase according to publication type (articles, reviews, and articles in press only). Subsequently, study eligibility was guided by the Population, Intervention, Comparator and Outcome (PICO) framework, as described in The Cochrane Handbook [1]. In addition, the review considered timing granularity, which was limited to annual data. The pre-defined PICO and complementary criteria for the studies that were included in this review are outlined in **Table S1.2**. All titles and abstracts identified by the search were screened, and PICO criteria were applied to assess eligibility. Duplicates in the Embase search results were also identified at this stage. A screening of the full text of all selected articles was performed by the same analyst using the pre-defined PICO criteria in **Table S1.2**. At both stages of the study selection (title/abstract, and full text), the project manager reviewed the abstracts of all excluded papers to ensure relevant articles were not omitted. PRISMA diagrams for the searches corresponding with the three data clusters are shown in following sections. Additional eligibility criteria are presented in **Table S1.3**.

| **Table S1.2** Data collection – PICO criteria. *GP* General practitioner; *HCW* Healthcare worker; *N/A* Not applicable; *PICO* Population, Intervention, Comparator and Outcome | | | | | |
| --- | --- | --- | --- | --- | --- |
| **PICO criteria** | | **Inclusion criteria** | | **Exclusion criteria** | |
| Population | | - Main focus - Older adults (aged ≥65 years, exceptions to this are dependent on countries) - Infants (children aged 6–23 months) - Pregnant women - HCWs - People with chronic conditions - Secondary focus if above not available - Children >2 years old - General population | | - Adolescents | |
| Intervention | | - Any - None | | - N/A | |
| Comparator | | - Any - None | | - N/A | |
| Outcome | | **Epidemiological input (including laboratory-confirmed, attributable to influenza, influenza-like illness)**   - Attack rate, GP consultations - Hospitalisation, intensive care unit admission, length of stay - Mortality | | - Pandemic influenza - Other infectious diseases related burden - Other health outcomes: Emergency visits, outbreaks, drug consumption - Other direct costs: Emergency visit, medication costs, non-direct costs (such as medical transport) - Disruption to the healthcare system - Vaccine effectiveness | |
|  | **Economic burden** • Direct costs: GP costs, hospital and intensive care unit admission costs (including complications) • Indirect costs: Absenteeism (lost workdays) | |  | |  |
|  | | | | | |

| Table S1.3 Data collection – Additional eligibility criteria. *NITAG* National Immunisation Technical Advisory Groups; *RCC* Regional Collaborating Committee; *VCR* Vaccination coverage rate; *WHO* World Health Organisation | | |
| --- | --- | --- |
| **Inclusion criteria** | | **Exclusion criteria** |
| Publication types | - **Published literature**: Scientific articles and reviews in peer-reviewed journals - **Official reports**: Epidemiological bulletins, surveillance reports, NITAG committee reports, Evidence from WHO RCC | - Research group reports, white papers, notes, short surveys, book chapters, tombstone - Conference proceedings: Abstracts, posters, expert presentations - Student thesis and other dissertations - Ongoing research: Unpublished manuscripts, work in progress academic papers - Press articles |
| Geographic granularity | - National basis | - Regional basis (except economic and VCR search) - Other geographies - Combined geographies |
| Time period | - Years of data collection: 2011/2012 to 2018/2019 season, most recent data preferred | - 2019/2020 season given COVID-19 pandemic - Pre-2011/2012 season |
| Timing granularity | - Seasonal basis | - Weekly basis - Monthly basis |
|  | | |

The screening process is illustrated in **Fig. S1.1**. These diagrams depict the search and screening process and show the number of studies excluded at each step. Thus, they provide summaries of the search methods and highlight the methodology in a transparent manner to ensure reproducibility of results.

After defining the targeted outcome for each epidemiological and economic input, a preferred source was selected for each country following a defined hierarchy of evidence (**Fig. S1.1**). Once studies were included following PICO criteria and PRISMA guidelines, they were ranked according to relevance. Where national health authority data, meta-analysis data, or literature review of homogeneous data were available, these were preferred as sources of data, thus reducing the number of studies from which data could be extracted. In a final step, studies with an extractable format, clear referencing, appropriate data stratification and representativeness were selected for extraction. In cases where all other parameters were similar, the study with the most recent data was chosen.

Fig. S1.1 Data collection **–** Detailed structuring of source selection and data extraction decisions for each systematic literature search (economic burden illustration). *OCEBM* University of Oxford Centre for Evidence-based Medicine; *PICO* Population Intervention Comparison Outcome; *PRISMA* Preferred Reporting Items for Systematic Reviews and Meta-Analyses; *RCT* randomised controlled trial; *SLR* systematic literature review


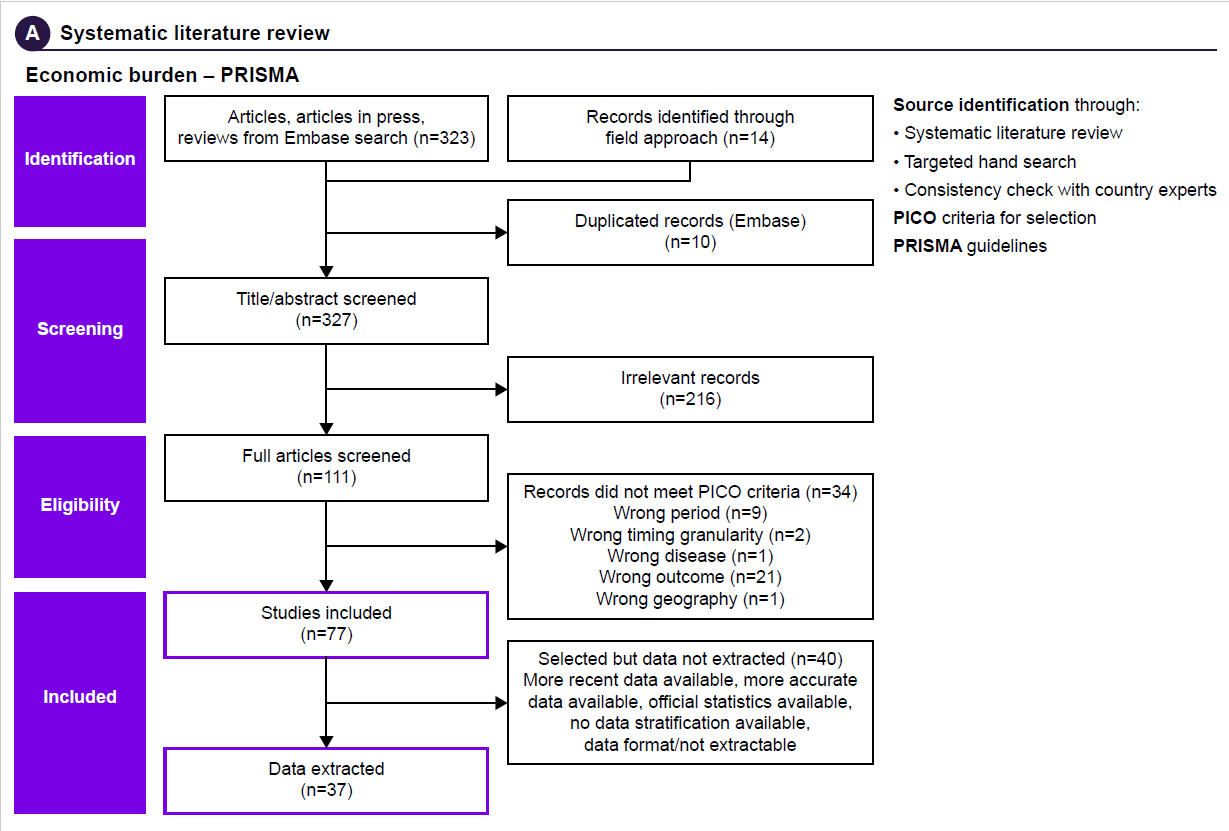


**
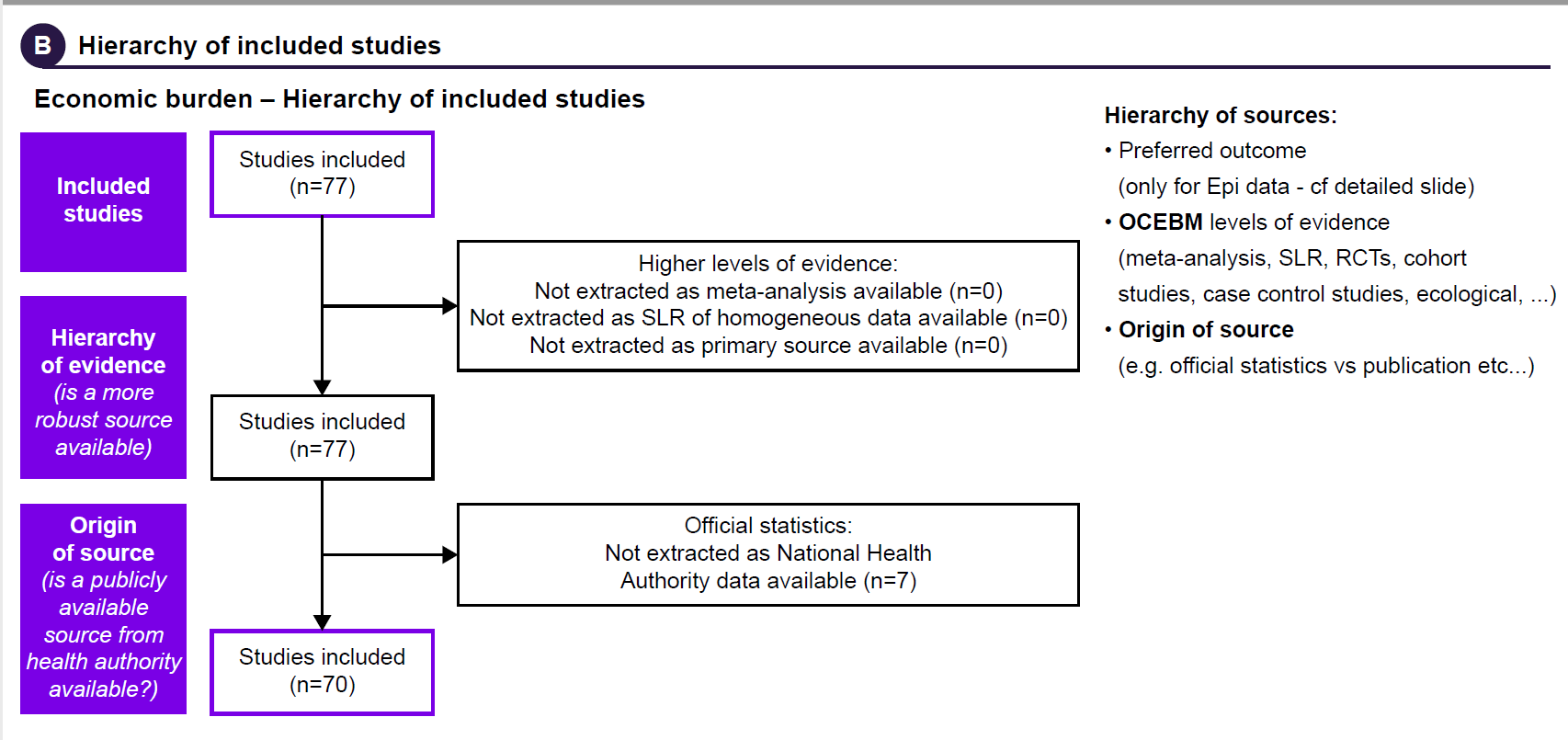
**

**
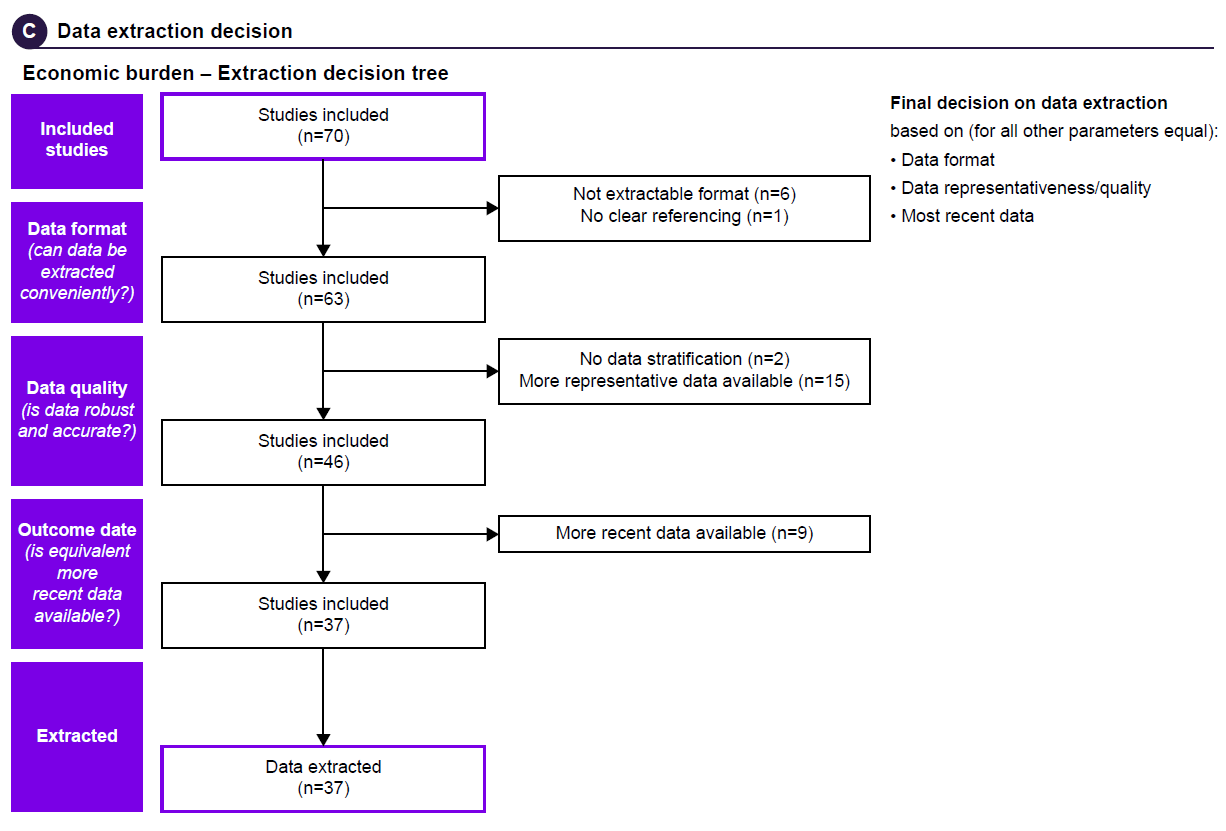
**

## VCR literature search

Multiple sources provide estimates of VCR data per risk group, such as national public health agencies, pan-European surveys, as well as clinical and behavioural studies that collected patients’ immunisation status.

A targeted literature review was performed to review the national public health agencies (Santé publique France [2], Ministero della Salute [3], Ministerio de Sanitad [4], Public Health England [5]) as well as relevant European sources, such as the European Centre for Disease Prevention and Control (ECDC) [6], the European Commission [7] and Eurostat [8].

When multiple sources of information were available, official statistics from the national public health agency were preferred, followed by statistics summarised by European institutions and finally data published by researchers.

Overall, most selected data were from national public health agencies, followed by data from European institutions.

## Public health burden literature search

Overall, 128 studies met the PICO criteria while the remaining studies were excluded as not matching these criteria. After a filter to remove studies in the same country where a preferred outcome is available, studies which are not the primary source of data or where national health authority data is available, 85 of the 128 studies were included. Finally, data were extracted from 44 studies. In particular, extracted values were expected to be expressed as rates (% or per 100,000 individuals), estimated on a national scale, covering several influenza seasons (ideally five to match the WHO recommendation for influenza disease burden estimates) and stratified per age groups and chronic condition status [9]. Data for rates of influenza-associated general practitioner (GP) visits, hospitalisation, and mortality were mostly available in France, Italy, Spain and the UK. Studies on all or one of the epidemiological inputs were found in a limited number of other countries. Data on excess GP visit rates were even scarcer with excess GP visit rate data identified in only one country (Germany), and excess hospitalisation and mortality data identified in six to seven countries.

## Economic impact literature search

The systematic literature review covered all countries in the scope of this study yielding approximately 460 scientific publications. Studies were screened according to PICO criteria resulting in 77 selected studies. A preference was then granted to national data when available, unless a global or European study was more relevant following the hierarchy of evidence. Both GP visit and hospitalisation costs were considered. Overall, GP costs and hospitalisation costs data were available in 16 countries.

# 2. Model inputs

## Population size inputs

According to World Health Organisation (WHO) and the age-based influenza recommendations for older adults in each country, demographic data were computed per 1‑year age group between 6 months and ≥90 years [10]. The 1-year age group demographics were populated using latest data available from Eurostat for France, Italy and Spain [11] and the Office for National Statistics (ONS) for the UK [12]. In France, Italy, Spain and the UK, the threshold for inclusion in the older adult age group is 65 years [13]; in these countries, influenza vaccinations are recommended for older adults.

For individuals with chronic conditions, data for each country were derived from Clark et al. [14] via exploratory analysis (see **Table S2.1**); specifically, the proportions of the population with at least one chronic condition were derived for males and females separately, per 5-year age group and per country. These demographic data were selected, as individuals at increased risk of severe disease tend to be similar for both influenza and COVID-19 [15]. These values were then applied to the population sizes from Eurostat. The sum of male and female individuals with chronic conditions aged ≥3 years over the country-specific recommended age group for vaccination of the older adults was then calculated. Individuals with chronic conditions were treated individually to avoid double-counting with those in the same risk group, but without underlying conditions.

| **Table S2.1** Percentage of individuals estimated to have chronic conditions***** according to sex as derived from Clark et al. [14] *Chronic conditions included: Human immunodeficiency virus/Acquired immunodeficiency syndrome, tuberculosis (excluding latent tuberculosis), cancers with direct immune suppression, cancers with possible immune suppression from treatment therapy, cardiovascular disease, chronic respiratory disease (excluding asthma), chronic liver disease, diabetes, chronic kidney disease, chronic neurological disorders, and sickle cell disorders. *UK* United Kingdom | | | | | | |
| --- | --- | --- | --- | --- | --- | --- |
| **Country** | **Age group (years)** | **Male (%)** | **Female (%)** | |  |  |
| France | 2–4 | 1.5 | 1.6 | |  |  |
|  | 5–9 | 2.1 | 2.1 | |  |  |
|  | 10–14 | 2.3 | 2.5 | |  |  |
|  | 15–19 | 3.7 | 4.4 | |  |  |
|  | 20–24 | 6.6 | 7.5 | |  |  |
|  | 25–29 | 9.4 | 10.5 | |  |  |
|  | 30–34 | 12.1 | 13.0 | |  |  |
|  | 35–39 | 15.3 | 15.1 | |  |  |
|  | 40–44 | 19.5 | 18.9 | |  |  |
|  | 45–49 | 24.3 | 22.5 | |  |  |
|  | 50–54 | 31.5 | 26.7 | |  |  |
|  | 55–59 | 42.0 | 33.9 | |  |  |
|  | 60–64 | 53.1 | 42.0 | |  |  |
| Italy | 2–4 | 1.5 | 1.4 | |  |  |
|  | 5–9 | 2.2 | 1.9 | |  |  |
|  | 10–14 | 2.3 | 2.4 | |  |  |
|  | 15–19 | 3.6 | 4.1 | |  |  |
|  | 20–24 | 6.6 | 7.4 | |  |  |
|  | 25–29 | 9.7 | 10.5 | |  |  |
|  | 30–34 | 12.8 | 13.3 | |  |  |
|  | 35–39 | 16.2 | 15.5 | |  |  |
|  | 40–44 | 20.2 | 19.0 | |  |  |
|  | 45–49 | 24.6 | 22.6 | |  |  |
|  | 50–54 | 31.7 | 26.8 | |  |  |
|  | 55–59 | 41.5 | 33.4 | |  |  |
|  | 60–64 | 52.2 | 41.6 | |  |  |
| Spain | 2–4 | 2.7 | 2.8 | |  |  |
|  | 5–9 | 3.4 | 3.3 | |  |  |
|  | 10–14 | 3.5 | 3.4 | |  |  |
|  | 15–19 | 4.9 | 5.0 | |  |  |
|  | 20–24 | 8.1 | 8.3 | |  |  |
|  | 25–29 | 11.1 | 11.3 | |  |  |
|  | 30–34 | 13.8 | 13.7 | |  |  |
|  | 35–39 | 17.0 | 15.9 | |  |  |
|  | 40–44 | 20.9 | 19.4 | |  |  |
|  | 45–49 | 25.9 | 23.0 | |  |  |
|  | 50–54 | 33.3 | 27.6 | |  |  |
|  | 55–59 | 42.7 | 34.2 | |  |  |
|  | 60–64 | 52.8 | 41.9 | |  |  |
| UK | 2–4 | 1.9 | 1.8 | |  |  |
|  | 5–9 | 3.0 | 2.7 | |  |  |
|  | 10–14 | 3.2 | 3.5 | |  |  |
|  | 15–19 | 4.8 | 6.4 | |  |  |
|  | 20–24 | 8.1 | 9.9 | |  |  |
|  | 25–29 | 11.2 | 13.5 | |  |  |
|  | 30–34 | 14.1 | 16.7 | |  |  |
|  | 35–39 | 17.1 | 19.0 | |  |  |
|  | 40–44 | 21.3 | 23.5 | |  |  |
|  | 45–49 | 26.7 | 28.1 | |  |  |
|  | 50–54 | 34.0 | 33.5 | |  |  |
|  | 55–59 | 44.0 | 41.2 | |  |  |
|  | 60–64 | 54.2 | 48.9 | |  |  |
|  | | | |  |  |  |

The number of pregnant women was assumed as equal to the number of live births extracted from Eurostat [16] for France, Italy and Spain and from ONS [17], National Records of Scotland [18] and Northern Ireland Statistics and Research Agency [19] for the UK, using the most recent data available. The population of pregnant women was then adjusted for chronic conditions using the proportion of 20­–40 year old females with chronic conditions from Clark et al. [14].

For healthcare workers (HCWs), data were extracted per HCW category from Eurostat [20] and the Organisation for Economic Co-operation and Development (OECD) [21] (practising doctors, nurses, midwives and healthcare assistants, as well as dentists, physiotherapists and pharmacists), using the most recently available data. Following the COVID-19 pandemic, the WHO Strategic Advisory Group of Experts (SAGE) issued a reminder of the importance of immunising healthcare personnel, highlighting HCWs as a higher priority risk group than older adults [10]. Beyond HCWs in the community, hospitals and nursing homes, the guidance recommends immunising workers in healthcare settings (e.g., outpatient staff, security and cleaning staff) next, if supply of immunisations permits. Furthermore, according to the 2017 ECDC report, the majority of EU countries recommend HCWs as a priority group for influenza vaccination. To avoid double counting, the population sizes for HCW and pregnant women were adjusted by excluding those with chronic conditions, using the proportion of adults aged 20–64 years with chronic conditions from Clark et al. [14]. The inputs used for the population are summarised in **Fig. S2.1** and in **Table S2.2.**


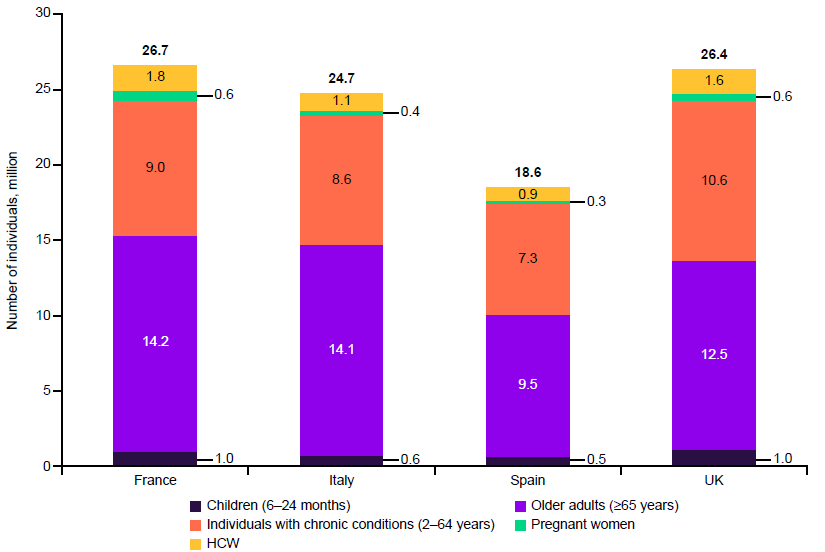
**Fig. S2.1** Model input – At-risk population size by country and risk groups for France, Italy, Spain and the UK. *HCW* Healthcare worker; *UK* United Kingdom. Data are presented to the nearest integer; discrepancies in totals are due to rounding.

| **Country** | **Children (6–24 months)** | **Older adults**  **(≥65 years)** | **Individuals with chronic conditions (2–17 years)** | **Individuals with chronic conditions (18–49 years)** | **Individuals with chronic conditions (50–59/ 64 years)** | **Pregnant women** | **HCWs** | **Total** |
| --- | --- | --- | --- | --- | --- | --- | --- | --- |

| France | 1,043,519 | | 14,225,883 | 324,525 | 3,689,459 | 4,951,994 | 649,868 | 1,774,558 | **26,659,805** |
| --- | --- | --- | --- | --- | --- | --- | --- | --- | --- |
| Italy | 607,947 | | 14,051,404 | 203,207 | 3,357,416 | 5,068,226 | 356,705 | 1,090,769 | **24,735,673** |
| Spain | 513,993 | | 9,526,631 | 269,635 | 3,134,461 | 3,938,014 | 297,828 | 904,619 | **18,585,180** |
| UK | 1,039,726 | | 12,537,031 | 415,987 | 4,617,349 | 5,547,996 | 590,760 | 1,632,021 | **26,380,870** |
| Total | **3,205,184** | | **50,340,949** | **1,213,353** | **14,798,685** | **19,506,230** | **1,895,161** | **5,401,966** | **96,361,528** |
|  | |  |  |  |  |  |  |  |  |

**Table S2.2** Model input – At-risk population size by country and risk groups for France, Italy, Spain and the UK. *HCW,* Healthcare worker;

*UK, United Kingdom*

## Vaccination coverage rate (VCR) inputs

The latest available country-specific VCR data for the 2021/2022 seasonal influenza season were collected for each WHO risk group. The data were sourced primarily from national public health agencies: Santé publique France [2], Ministero della Salute [3], Ministerio de Sanidad [4], Public Health England [5], and country-specific data from the ECDC database. This was complemented [22] by a targeted literature review (see section Data collection and classification of evidence), in particular for individuals with chronic conditions (Italy and Spain) and the paediatric population (France, Italy and Spain) [18]. The vaccination gap between the WHO-recommended 75% VCR target and the eligible vaccinated population in 2021/2022 was calculated for each country.

The targeted literature research yielded VCR data from all countries for all the risk groups. Official sources were available for 17 of the 25 VCR. For the remaining eight VCR, publications with relevant data were included. Finally selected data, including relevant year and sources are presented in the tables below.

**Table S2.3** Model inputs – VCR by age and risk group for EU-5 countries (i.e., United Kingdom, Germany, France, Spain, Italy). *ECDC* European Centre for Disease Prevention and Control*; PHE* Public Health England; *UK*, United Kingdom*; VCR,* vaccination coverage rate;

| **Table S2.3** A: Children (aged <18 years) | | | | |
| --- | --- | --- | --- | --- |
| **Country** | **VCR** | **Season** | **Sources** |  |
| France | 5% | 2009–2010 | Vaux et al., 2011 [23] |  |
| Italy | 7% | 2021–2022 | Ministero della Salute 2022 [3] |  |
| Spain | 7% | 2005–2007 | Lopez-de-Andres et al., 2009 [24] |  |
| UK | 0% | 2021–2022 | England data, PHE 2022 [5] |  |
| **Table S2.3** B: Older adults (aged ≥65 years) | | | | |
| **Country** | **VCR** | **Season** | **Sources** |  |
| France | 57% | 2021–2022 | Santé publique France 2023 [25] |  |
| Italy | 58% | 2021–2022 | Ministero della Salute 2022 [3] |  |
| Spain | 69% | 2021–2022 | Comité Asesor de Vacunas de la AEP 2022 [26] |  |
| UK | 82% | 2021–2022 | England data, PHE 2022 [5] |  |

| **Table S2.3** C: Individuals with chronic conditions*. *Chronic conditions included: diabetes mellitus and other metabolic diseases, chronic cardiopathy, cancer, chronic lung diseases, chronic inflammatory diseases and bowel malabsorption syndromes, pathologies associated with an increased risk of aspiration of respiratory secretions, chronic respiratory disease, chronic liver diseases, chronic neurological disease (including stroke/ transient ischaemic attack, cerebral palsy/multiple sclerosis), chronic renal failure/adrenal insufficiency, human immunodeficiency virus and immunodepression, haematopathies and haemoglobinopathies, asplenia or dysfunction of the spleen, morbid obesity, and pathologies for which major surgical interventions are planned. | | | | |
| --- | --- | --- | --- | --- |
| **Country** | **VCR** | **Season** | **Sources** |  |
| France | 34% | 2021–2022 | Santé publique France 2022 [27] |  |
| Italy | 29% | 2020–2021 | Bianchi et al., 2023 [28] |  |
| Spain | 17% | 2011–2012 | Mochales et al., 2017 [29] |  |
| UK | 53% | 2021–2022 | England data, PHE 2022 [5] |  |

| **Table S2.3** D: Pregnant women | | | | |
| --- | --- | --- | --- | --- |
| **Country** | **VCR** | **Season** | **Sources** |  |
| France | 8% | 2021–2022 | Santé publique France 2022 [27] |  |
| Italy | 2% | 2016–2017 | ECDC [13] |  |
| Spain | 55% | 2021–2022 | Comité Asesor de Vacunas de la AEP 2022 [26] |  |
| UK | 38% | 2021–2022 | England data, PHE 2022 [5] |  |

| **Table S2.3** E: Healthcare Workers | | | | |
| --- | --- | --- | --- | --- |
| **Country** | **VCR** | **Season** | **Sources** |  |
| France | 22% | 2021–2022 | Santé publique France 2022 [27] |  |
| Italy | 16% | 2016–2017 | ECDC [13] |  |
| Spain | 61% | 2021–2022 | Comité Asesor de Vacunas de la AEP 2022 [26] |  |
| UK | 61% | 2021–2022 | England data, UK Health Security Agency 2022 [30] |  |
|  | | | | |

## Epidemiological inputs

A 2018 meta-analysis from Somes et al. was selected as the source for influenza attack rates [31]. It included 32 randomised controlled trials and provides attack rates for the general unvaccinated population, stratified by age. Reported attack rates were 4.4% (95% confidence interval [CI]: 3.0–6.3) for adults, 7.2% (95% CI: 4.3–12.0) for older adults (≥65 years) and 12.7% (95% CI: 8.5–18.6) for children (<18 years) [31].

GP visit rates for influenza were found in three countries (France, Spain and the UK) based on the review of the scientific literature and national influenza surveillance systems. The UK was the only country producing inputs for individuals with chronic conditions and all-population inputs [32]. In two countries (Spain and France), the average influenza GP visit rates for each population over several seasons was taken (2013–2019 for Spain and 2014/2015–2018/2019 for France); the age stratification was missing. Age stratifications used in this analysis were therefore extrapolated from influenza hospitalisation data reported in ISCII reports for Spain and Lemaitre et al. for France [33, 34].

Given the lack of country-specific data on influenza GP visit rates in the chronic population, a risk factor of 1.51 was applied on data from the general population based on a publication from Baguelin et al. [35], that estimated the difference in influenza-related GP visits between the healthy and the chronic population. A key limitation is that this risk factor was applied to GP visit rates estimated on the general population, thus leading to an overestimate of the results as the general population already included chronic population. Therefore, the subsequent deterministic analysis also included an evaluation of the impact on the results were this risk factor not applied.

For Italy, the GP visit rates for acute respiratory infections were identified, thus requiring an adjustment by a positivity ratio to evidence the actual share attributable to influenza. A recent European meta-analysis from Belazi et al. [36], published in Eurosurveillance estimated the ratio to be 0.26 for individuals aged 0–17 years, 0.41 for individuals aged 18–64 years, and 0.33 for individuals aged ≥65 years.

While there may be ≥1 visit made by a patient (i.e. a patient may have a second visit in a case of complications), an assumption of one visit per patient was taken for simplification purposes. Thus, the number of GP visits may be underestimated, since Carrat et al. estimated the number of visits at 1.35 per case in France [37].

| Table S2.4 Literature review – Influenza GP visit rates derived from literature review (per 100,000). *Individuals with chronic conditions. *GP* General practitioner; *ISCII* Instituto de Salud Carlos III; *HCW* Healthcare worker; *UK* United Kingdom | | | | | | | | | | | | | | | | | | | | | | | | | | | | | | |  |  |
| --- | --- | --- | --- | --- | --- | --- | --- | --- | --- | --- | --- | --- | --- | --- | --- | --- | --- | --- | --- | --- | --- | --- | --- | --- | --- | --- | --- | --- | --- | --- | --- | --- |
| **Country** | **Children (6–24 months)** | | | **Sources** | **Older adults (≥65 years)** | | **Sources** | | | **Individuals* (2–17 years)** | | | | **Sources** | **Individuals* (18–49 years)** | | | **Sources** | | **Individuals* (50–59/ 64 years)** | | **Sources** | | **Pregnant women** | | **Sources** | **HCWs** | | | **Sources** | |  |
| France | | 4,681 | | Paternos ter et al. [38] (2014/15–2018/19 mean) | 988 | | Paternos ter et al. [38] (2014/15–2018/19 mean) | | | 6,375 | | | | Paternos ter et al. [38] (2014/15–2018/19 mean, age-groups; weighted average), Baguelin et al. [35] | 3,532 | | | Paternos ter et al. [38] (2014/15–2018/19 mean), Baguelin et al. [35] | | 3,532 | | Paternos ter et al. [38] (2014/15–2018/19 mean), Baguelin et al. [35] | | 2,339 | | Paternos ter et al. [38], (2014/15–2018/19 mean; aged 18–49 years, non- chronic conditions) | 2,339 | | | Paternos ter et al. [38] (2014/15–2018/19 mean; aged 18–49 and  50–64 years, non- chronic conditions; weighted average) | |  |
| Italy | | | 1,378 | Mennini et al. [39], Belazi et al. [36], | | 314 | | Mennini et al. [39], Belazi et al. [36], | 1,593 | | | Mennini et al. [39], Belazi et al. [36], Baguelin et al. [35] | | | 1,139 | | Mennini et al. [39], Belazi et al. [36], Baguelin et al. [35] | | 1,068 | | Mennini et al. [39], Belazi et al. [36], Baguelin et al. [35] | | 755 | | | Mennini et al. [39], Belazi et al. [36] (aged 18–49 years, non-chronic conditions) | 737 | | Mennini et al. [39], Belazi et al. [36] (aged 18–49 and 50–64 years, non-chronic conditions; weighted average) | | | |
| Spain | | | 4,790 | ISCII reports [33] (mean 2013/14–2019/20) | | 797 | | ISCII reports [33] (mean 2013/14–2019/20, age-group; weighted average) | | | 5,401 | | ISCII reports [33] (mean 2013/14–2019/20), Baguelin et al. [35] | | | 2,449 | ISCII reports [33] (mean 2013/14–2019/20), Baguelin et al. [35] | | 2,449 | | ISCII reports [33] (mean 2013/14–2019/20), Baguelin et al. [35] | | 1,622 | | | ISCII reports [33] (18–49 non chronic, mean 2013/14–2019/20) | 1,622 | | ISCII reports [33]  (mean 2013/14–2019/20; aged 18–49 and 50–64 years, non-chronic conditions; weighted average,) | | | |
| UK | | | 753 | Fleming et al. [40] (influenza A and B) | | 307 | | Fleming et al. [40] (influenza A and B) | | | 763 | | Fleming et al. [40] (influenza A and B for high-risk people, age-groups; weighted average) | | | 636 | Fleming et al. [40] (influenza A and B for high-risk people) | | 537 | | Fleming et al. [40] (influenza A and B for high-risk people) | | 449 | | Fleming et al. [40] (aged 18–49 years, non-chronic conditions, influenza A and B) | | | 442 | Fleming et al. [40] (aged 18–49 and  50–64 years, non-chronic conditions; weighted average, influenza A and B) | | | |
|  | | | | | | | | | | | | | | | | | | | | | | | | | | | | | | |  |  |

Finally, all GP visit rates were adjusted to include vaccination-neutral rates i.e., GP visit rates which were unrelated to vaccinations were included within the adjusted rates. A systematic approach was followed to ensure consistency of the GP visit rate inputs, using the latest VCR available (c.f. **Table S2.3**) and the vaccine efficacy of this study (c.f. **Table S2.17**). The following formula was used: (${GP visit rate}_{used}= \frac{{GP visit rate}_{input}}{1-VCR\times(Vaccine efficacy)}$). The inputs for influenza-related GP visits, estimated based on an extrapolated analysis, are provided in **Table S2.5**.

| **Country** | **Children (6–24 months)** | | **Older adults (≥65 years)** | **Individuals with chronic conditions  (2–17 years)** | **Individuals with chronic conditions (18–49 years)** | **Individuals with chronic conditions (50–64 years)** | **Pregnant women** | **HCWs** |
| --- | --- | --- | --- | --- | --- | --- | --- | --- |
| France | 5,019 | | 1,499 | 8,277 | 4,466 | 4,466 | 2,459 | 2,702 |
| Italy | 5,019 | | 481 | 1,972 | 1,381 | 1,295 | 762 | 814 |
| Spain | 1,446 | | 1,365 | 6,119 | 2,742 | 2,742 | 2,440 | 2,571 |
| UK | 755 | | 606 | 1,182 | 939 | 793 | 584 | 701 |
|  | |  |  |  |  |  |  |  |

**Table S2.5** Model input – Estimated influenza-related GP visits per 100,000. *HCW,*

Healthcare worker

Excess hospital admissions stratified by age group were extracted from the literature for the four countries. Respiratory excess hospital admission rates were used for France [34], Italy [41], and the UK [42] and cardiorespiratory excess hospital admission rates were used for Spain [43]. The results are shown in **Table S2.6**.

As no meta-analyses or systematic literature reviews of influenza-associated excess hospitalisation rates in Europe were found, the manual search was expanded to global studies. Given the lack of data on patients with chronic conditions, a risk factor was applied to estimate the rates to be used as input for the chronic risk group. A recent meta-analysis (Coleman et al. [44]) found that patients with chronic conditions were 2.25 times more likely to be hospitalised than people without chronic conditions. This risk factor was applied to the rates from the general population as rates only including the healthy population were not available, thus leading to an overestimation of results as the general population already included the chronic population. Therefore, the subsequent deterministic analysis also included an evaluation of the impact on the results if this risk factor were not applied.

**Table S2.6** Literature review – influenza-associated excess hospitalisation rates extracted from literature (per 100,000). *HCW* Healthcare worker; *UK* United Kingdom

|  |  | |  | |  |  |  |  |  |  |  |  |  |  |  |  |
| --- | --- | --- | --- | --- | --- | --- | --- | --- | --- | --- | --- | --- | --- | --- | --- | --- |
| **Country** | **Children (6–24 months)** | | **Sources** | | **Older adults (≥65 years)** | **Sources** | **Individuals with chronic conditions  (2–17 years)** | **Sources** | **Individuals with chronic conditions (18–49 years)** | **Sources** | **Individuals with chronic conditions (50–64 years)** | **Sources** | **Pregnant women** | **Sources** | **HCWs** | **Sources** |
| France | 22.7 | | Lemaitre et al. [34] (mean 2010–17), Gil et al. age group stratification [43] | | 183.6 | Lemaitre et al. [34] (mean 2010–17) | 26.0 | Lemaitre et al. [34] (mean 2010–17), age groups; weighted average), Gil et al. age group stratification [43], Coleman et al. [44] | 20.1 | Lemaitre et al. [34] (mean 2010–17), age groups; weighted average, Gil et al. age group stratification [43], Coleman et al. [44] | 83.6 | Lemaitre et al. [34] (mean 2010–17), Gil et al. age group stratification [43], Coleman et al. [44] | 8.9 | Lemaitre et al. [34] (mean 2010–17), Gil et al. age group stratification [43], Coleman et al. [44] (18–49 non chronic) | 18.3 | Gil et al. age group stratification [43], Coleman et al. [44] (18–49 and 50–64, patients with non-chronic conditions; weighted average) |
| Italy | 27.0 | | Bertolani et al. [41] (mean 2008–15), Gil et al. age group stratification [43] | | 105.8 | Bertolani et al. [41] (mean 2008–15), Gil et al. age group stratification [43] | 69.8 | Bertolani et al. [41] (mean 2008–15, age groups weighted average), Gil et al. age group stratification [43], Coleman et al. [44] | 12.0 | Bertolani et al. [41] (mean 2008–15), Gil et al. age group stratification [43], Coleman et al. [44] | 41.8 | Bertolani et al. [41] (mean 2008–15), Gil et al. age group stratification [43], Coleman et al. [44] | 5.3 | Bertolani et al. [41] (mean 2008–15), (aged 18–49 years; non chronic conditions) | 10.4 | Bertolani et al. [41] (mean 2008–15), (aged 18–49 and 50–64; non chronic conditions; weighted average) |
| Spain | 19.5 | | Gil et al. [43] mean 2008/09–2017/18 | | 335.3 | Gil et al. [43] mean 2008/09–2017/18 | 39.8 | Gil et al. [43] mean 2008/09–2017/18, age-groups weighted average, Coleman et al. [44] | 24.3 | Gil et al. [43] mean 2008/09–2017/18, Coleman et al. [44] | 123.3 | Gil et al. [43] mean 2008/09–2017/18, Coleman et al. [44] | 10.8 | Gil et al. [43] mean 2008/09–2017/18, 18–49 non chronic | 26.1 | Gil et al. [43] (mean 2008/09–2017/18, 18–49 and 50–64 non chronic weighted average) |
| UK | 93.0 | | Matias et al. [42] | | 171.3 | Matias et al. [42] (age groups; weighted average) | 0.8 | Matias et al. [42] (age groups; weighted average) | 65.0 | Matias et al. [42] (age groups; weighted average) | 124.0 | Matias et al. [42] (age groups; weighted average) | 28.9 | Matias et al. [42] (age groups; weighted average, aged 18–49 years, non-chronic conditions) | 37.4 | Matias et al. [42] (age groups; weighted average, aged 18–49 and 50–64 years, non-chronic conditions) |
|  | |  | |  |  |  |  |  |  |  |  |  |  |  |  |  |

When the age-specific excess hospitalisation rates were not available for the population with chronic conditions (France and Italy), the age-specific rate was obtained using the Gil et al. [43] age stratification for cardiorespiratory influenza-associated hospitalisations (2008–2018; the proportion of total hospitalisations which patients in each age group accounted for was calculated). The age-stratification used is shown in **Table S2.7**.

| Table S2.7 Model input – Gil et al. [43], Spain age distribution for influenza-associated excess hospitalisation rates | | |
| --- | --- | --- |
| **Age group (years)** | **Weight of the group in hospitalisations** |  |
| <5 | 3% |  |
| 5–18 | 2% |  |
| 19–49 | 6% |  |
| 50–64 | 13% |  |
| ≥65 | 76% |  |

Finally, all hospitalisation rates were adjusted to include vaccination-neutral rates i.e., hospitalisation rates which were unrelated to vaccinations were included within the adjusted rates. A systematic approach was followed to ensure consistency of the hospitalisation rate inputs, using the latest VCR available (c.f. **Table S2.3**) and the Vaccine efficacy of this study (c.f. **Table S2.17**). The following formula was used:

($=$)

The full inputs for excess influenza-associated hospitalisations are provided in **Table S2.8**.

| Table S2.8 Model input – Estimated influenza-associated excess hospitalisation rates per 100,000. *HCW* Healthcare worker; *UK* United Kingdom | | | | | | | | | | | | | | |  |
| --- | --- | --- | --- | --- | --- | --- | --- | --- | --- | --- | --- | --- | --- | --- | --- |
| **Country** | | **Children (6–24 months)** | | | | **Older adults (≥65 years)** | | | **Individuals with chronic conditions (2–17 years)** | **Individuals with chronic conditions (18–49 years)** | **Individuals with chronic conditions (50–64 years)** | **Pregnant women** | **HCWs** |  |  |
| France | | | 20.4 | | | | 278.5 | | 33.7 | 25.4 | 105.7 | 9.4 | 21.2 |  |  |
| Italy | | | 28.3 | | | | 162.5 | | 86.4 | 14.5 | 50.7 | 5.4 | 11.4 |  |  |
| Spain | | | 5.3 | | | | 574.5 | | 45.1 | 27.2 | 138.0 | 16.2 | 41.3 |  |  |
| UK | 20.4 | | | | 338.4 | | | 1.3 | | 96.0 | 183.1 | 37.6 | 59.3 |  |  |
|  | | | |  | | | | | | | | | | | |

The preferred outcome values on all-cause excess mortality attributable to influenza was found at European and national levels over several influenza seasons and stratified by age; however, no values were found for the chronic population. Consistent with Preaud et al. [45], all deaths occurring in the 2–64 year-old patients/population(s)/age groups(s) were assumed to have occurred in the chronic population, while no influenza mortality was assumed in healthy pregnant women and HCWs.

Country-specific data from literature were used for Italy [46], Spain [47], and the UK [48] for all the target groups. For France, country-specific older adults excess mortality rate was used [34], whereas European all-cause influenza-attributable values were used for individuals with chronic conditions, pregnant women, children and HCWs from Nielsen et al. [49], which is the most comprehensive European study, covering 18 countries (24 territories) over five influenza seasons (from 2012/2013–2017/2018) and providing rates stratified to four age groups. The average yearly excess mortality rate associated with influenza-related mortality and its CIs were estimated as the mean of multiple seasons. The inputs for national excess influenza-associated mortality rates are presented in **Table S2.9**.

To extrapolate the mortality rates for the chronic population, it was assumed that all influenza-associated deaths occurred in the chronic-conditions population.

**Table S2.9** Literature review – all-cause excess mortality rates per 100,000 for France, Italy, Spain and the UK extracted from literature. *PHE* Public Health England; *UK* United Kingdom

| **Country** | **Children (6–24 months)** | | **Sources** | **Older adults**  **(≥65 years)** | **Sources** | **Individuals with chronic conditions  (2–17 years)** | **Sources** | **Individuals with chronic conditions (18–49 years)** | **Sources** | **Individuals with chronic conditions (50–64 years)** | **Sources** |  |
| --- | --- | --- | --- | --- | --- | --- | --- | --- | --- | --- | --- | --- |
| France | 0.7 | | Nielsen et al. [49], 2012/13–2017/18 mean | 81.58 | Lemaitre et al. [34] (mean 2010/11–2014/15) | 24.0 | Nielsen et al. [49], 2012/13–2017/18 mean | 14.3 | Nielsen et al. [49], 2012/13–2017/18 mean | 5.3 | Nielsen et al. [49], 2012/13–2017/18 mean |  |
| Italy | 0.7 | | Rosano et al. [46] mean 2013–17 | 108.14 | Rosano et al. [46] mean 2013–17 | 32.0 | Rosano et al. [46] mean 2013–17, age-groups weighted average | 16.2 | Rosano et al. [46] mean 2013–17 | 6.6 | Rosano et al. [46] mean 2013–17 |  |
| Spain | 1.1 | | Gil et al. [47] mean 2012/13–2017/18 | 133.53 | Gil et al. [47] mean 2012/13–2017/18 | 6.5 | Gil et al. [47] mean 2012/13–2017/18, age-groups weighted average | 5.3 | Gil et al. [47] mean 2012/13–2017/18, age-groups weighted average | 24.9 | Gil et al. [47] mean 2012/13–2017/18 |  |
| UK | 1.5 | | PHE FluMOMO [48] | 116.33 | PHE FluMOMO [48] | 21.2 | PHE FluMOMO [48], age groups weighted average | 11.6 | PHE FluMOMO [48] | 4.64 | PHE FluMOMO [48] |  |
|  | |  |  |  |  |  |  |  |  |  |  |  |

Finally, mortality rates were adjusted to include vaccination-neutral mortality rates i.e., mortality rates which were unrelated to vaccinations were included within the adjusted rates. A systematic approach was followed to ensure consistency of the mortality rate inputs, using the latest VCR available (c.f. **Table S2.3**) and the vaccine efficacy of this study (c.f. **Table S2.17**). The following formula was:

$$=$$

The model inputs for all-cause excess mortality rates are shown in **Table S2.10**.

**Table S2.10** Model input – Estimated all-cause excess mortality rates per 100,000. *UK* United Kingdom

| **Country** | **Children (6–24 months)** | **Older adults (≥65 years)** | **Individuals with chronic conditions  (2–17 years)** | **Individuals with chronic conditions (18–49 years)** | **Individuals with chronic conditions (50–64 years)** |
| --- | --- | --- | --- | --- | --- |
| France | 1.19 | 123.76 | 31.18 | 18.02 | 6.72 |
| Italy | 0.78 | 166.02 | 39.56 | 19.67 | 7.99 |
| Spain | 1.19 | 228.80 | 7.33 | 5.95 | 27.85 |
| UK | 1.46 | 229.81 | 32.77 | 17.16 | 6.84 |

A further consequence of influenza is the incapacitation of patients leading to an inability to work. The number of preventable days missed from work due to influenza disease for adults was estimated using the following formula:

*Preventable Lost Workdays _adult. country_ = Preventable GP consultations _adult. country_ x Average Days Missed _adult. country_ x Employment Rate _country_*

The average days missed from work was calculated as:

*Average Days Missed _adult. country_ = Probability of taking a sick leave _adult. country_ x Workdays lost per sick leave _adult. country_*

A literature review was initially performed to obtain values for the countries considered. While several studies and data points were identified, the heterogeneity of data was such that selecting one set of values from the most robust available studies and then applying it to all countries was used instead. Scholz et al. [50] analysed claims data from 2012 to 2014 in Germany comprising >8 million insured members by calculating the proportion of insured members who were on sick leave from work due to influenza (confirmed diagnosis). The available data for days of work missed is conditional of employment and sick leave status. The expected number of days taken off work for adults in this study was thus estimated as the product of a 54.9% probability of taking time off for sickness from influenza, conditional to GP visits and the average duration of time off for influenza sickness, this was taken as 6.87 days with both values from Scholz et al. [50]. This was applied to all countries and for all adult target groups, except HCWs, given their higher risk of exposure to sick patients and increased requirements to avoid work if infected. For HCWs, the expected number of sick days was estimated based on a 54.9% probability of taking time off for sickness due to influenza, and the average duration of time off for influenza sickness (7.2 days) [51]. This was applied to all countries and for all HCWs.

Additionally, sickness in children might require a parent to take time off work to care for their child. In this study, the number of preventable days missed from work for a parent were estimated with the following formula:

*Preventable Lost Workdays _parent. country_ =Preventable GP consultations _child. country_ x Average Days Missed _parent. country_ x Employment Rate _country_*

Here the average days missed from work were calculated as

*Average Days Missed _parent. country_ = Probability of taking a sick leave _parent. country_ x Workdays lost per sick leave _parent. country_*

The expected number of days off work for parents in this study was thus estimated as the product of a 25% probability of parents taking time off (due to their child having influenza), and the average duration of parental time off as 3 days [52].

To remain conservative, no influenza-associated absenteeism was estimated among older adults. However, a proportion of seniors were still working beyond the age of 65 and could miss work because of influenza. The results are shown in **Table S2.11**.

| Table S2.11 Model input – average days missed from work conditional on sick leave for influenza for the employed population. *HCW* Healthcare worker | | | | | | | |  |
| --- | --- | --- | --- | --- | --- | --- | --- | --- |
| **Country** | **Older adults (≥65 years)** | **Individuals with chronic conditions  (2–17 years)** | **Individuals with chronic conditions (18–49 years)** | **Individuals with chronic conditions (50–64 years)** | **Pregnant women** | **Children (6–24 months)** | **HCWs** | |
| Workdays lost | – | 3.00 | – | – | – | 3.00 | – | |
| Calendar days lost | – | – | 6.87 | 6.87 | 6.87 | – | 7.20 | |
| Probability | – | 25% | 55% | 55% | 55% | 25% | 55% | |
| Average Days | – | 0.75 | 3.77 | 3.77 | 3.77 | 0.75 | 3.95 | |
| Source | – | Gerlier et al. 2017 [52] | Scholz et al. 2019 [50] | Scholz et al. 2019 [50] | Scholz et al. 2019 [50] | Gerlier et al. 2017 [52] | Diel et al. 2019 [51], Scholz et al. 2019 [50] | |
|  | | | | | | | |  |

The number of workdays avoided based on hospital cases was then added to compute the total number of days missed at work. The 2010–2018 mean influenza hospital length of stay from Lemaitre et al. [34], was used as a proxy for sick leave duration for all the countries with data for 0–4 years, 5–19 years, 20–49 years, 50–64 years, 65–84 years and ≥85 years. A weighted average of the influenza hospital length of stay was calculated in the HCW population based on population sizes to obtain data points for these specific age groups that are not shown in Lemaitre et al. [34]. These length of stay inputs are shown in the **Table S2.12**.

| Table S2.12 Model input – Length of Stay due to influenza hospitalisation. *HCW* Healthcare worker; *LOS* Length of stay | | | | | | | |
| --- | --- | --- | --- | --- | --- | --- | --- |
|  | **Older adults (≥65 years)** | **Individuals with chronic conditions  (2–17 years)** | **Individuals with chronic conditions (18–49 years)** | **Individuals with chronic conditions (50–64 years)** | **Pregnant women** | **Children (6–24 months)** | **HCWs** |
| Influenza hospital LOS – median | 8.4 | 3.0 | 4.0 | 7.0 | 4.0 | 3.0 | 5.0 |
| Influenza hospital LOS – minimum | 7.4 | 3.0 | 3.0 | 6.0 | 3.0 | 3.0 | 4.0 |
| Influenza hospital LOS – maximum | 9.2 | 3.0 | 4.0 | 7.0 | 4.0 | 3.0 | 5.0 |
| Source | Lemaitre et al. 2021 [34], Age-group weighted average | Lemaitre et al. 2021 [34] | Lemaitre et al 2021. [34] | Lemaitre et al. 2021 [34] | Lemaitre et al. 2021 [34] | Lemaitre et al. 2021 [34] | Lemaitre et al. 2021 [34], Age-group weighted average |
|  | | | | | | | |

The number of workdays missed due to influenza hospital stay was then computed using the following formula:

*Number of days missed due to an influenza hospital stay = Number of hospitalisations avoided x Employment rate* *x Influenza hospital length of stay*

## Cost inputs

GP visit costs for influenza-associated visits were country-specific. GP costs for adults were identified in all four countries and for children only in France. When the value for children is not available, the adults GP cost was taken for children. Collected GP visit costs can be specific to an influenza consultation or be a standard rate used for any type of consultation, dependent on the country.

GP visit unitary costs were used for France, Italy, Spain and the UK and the costs related to GP visits are therefore underestimated.

All literature values were converted from local currency into euros, and an inflation rate was applied when relevant [53, 54]. GP visits costs inputs are shown in **Table S2.13**.

| Table S2.13 Costs of GP visits per country – Standardised at 2022 EUR. *CPI* Consumer price index; *GP* General practitioner; *HCW* Healthcare worker; *OECD* Organisation for Economic Co-operation and Development; *UK* United Kingdom | | | | | | | | | | |  |
| --- | --- | --- | --- | --- | --- | --- | --- | --- | --- | --- | --- |
| **Country** | **Children**  **(6–24 months)** | | **Older adults**  **(≥65 years)** | **Individuals with chronic conditions  (2–17 years)** | **Individuals with chronic conditions (18–49 years)** | **Individuals with chronic conditions (50–64 years)** | **Pregnant women** | **HCWs** | **Sources** |  |  |
| France | €30 | | €25 | €25 | €25 | €25 | €25 | €25 | “Tarifs des consultations du médecin traitant (03 octobre 2022)“. taken from Ameli.fr [55] |  |  |
| Italy | €23 | | €23 | €23 | €23 | €23 | €23 | €23 | Capri et al. 2018 [56]. CPI adjusted (OECD statistics) [53] |  |  |
| Spain | €69 | | €69 | €69 | €69 | €69 | €69 | €69 | Gil et al. [43] |  |  |
| UK | €61 | | €61 | €61 | €61 | €61 | €61 | €61 | Hodgson et al. 2017 [57]. Value adjusted for CPI (OECD statistics) [53] and converted in euros [54] |  |  |
|  | |  | | | | | | | | | |

Hospitalisation unitary costs were available for the four countries from Diagnosis Related Group tariffs, hospitalisation statistics, and published literature. While there was variation between countries in the national or international codes used, the code definitions were similar enough to justify the use of country-specific data. All values were converted from local currency into euros and an inflation rate was applied based on consumer price index [53, 54]. The hospitalisation costs inputs are shown in **Table S2.14**.

| Table S2.14 Estimated Costs of hospitalisation per country – Standardised at 2022 EUR. *CPI* Consumer price index; *GP* General practitioner; *HCW* Healthcare worker; *OECD* Organisation for Economic Co-operation and Development; *UK* United Kingdom | | | | | | | | | |  |
| --- | --- | --- | --- | --- | --- | --- | --- | --- | --- | --- |
| **Country** | **Children**  **(6–24 months)** | **Older adults**  **(≥65 years)** | **Individuals with chronic conditions  (2–17 years)** | **Individuals with chronic conditions (18–49 years)** | **Individuals with chronic conditions (50–64 years)** | **Pregnant women** | **HCWs** | **Sources** |  |  |
| France | €2,100 | €6,018 | €3,522 | €3,808 | €3,808 | €3,808 | €3,808 | Lemaitre et al. 2021 [34] and adjusted for CPI (OECD statistics) [53] |  |  |
| Italy | €3,422 | €3,742 | €3,422 | €3,742 | €3,742 | €3,742 | €3,742 | Boccalini et al. 2018 [58], Dal Negro et al. 2018 [59]. Value adjusted for CPI (OECD statistics) [53] |  |  |
| Spain | €2,614 | €3,964 | €3,002 | €4,915 | €5,394 | €4,915 | €5,464 | Gil et al. 2021 [43] |  |  |
| UK | €2,512 | €4,418 | €2,521 | €2,521 | €2,521 | €2,521 | €2,521 | Moss et al. 2020 [60]. Value converted in Euros [54] and adjusted for CPI (OECD statistics) [53] |  |  |
|  |  | | | | | | | | | |

To estimate the value of avoidable lost wages, the cost per day of missed work was computed. To obtain this information these steps were followed:

1) From OECD 2021 statistics [61], the value of average annual gross wages per full-time employee was obtained.

2) The average number of working days per country was obtained for children (6–24 months), older adults (≥65 years) and individuals 3–17 years old with chronic conditions using the following formula:

Working days _country_ = 52*5 –length of vacation _country_ – Public Holidays _country_

In other words, the length of statutory minimum vacation was subtracted from the full year adjusted by the number of vacation days, as well as the number of public holidays per country.

For individuals 18–49 years old and 50–64 years old, with chronic conditions, pregnant woman and HCWs, we based our computation on the 365 calendar days as the study used is a German one (sick leave duration is based on calendar days in Germany).

3) Next, the country-specific average annual wage per worker was divided by the number of working days for older adults (≥65), individuals 3–17 years old with chronic conditions, and children (6–24 months), and by the number of calendar days for individuals 18–49 years old with chronic conditions, individuals 50–64 years old with chronic conditions, pregnant women and HCWs to obtain the average costs of a sick day per country. Gross wages inputs are shown in **Table S2.15**.

4) Finally, to estimate the total value of work-related monetary losses that can be prevented by increasing VCR to 75%, or for the other scenarios, the number of potentially preventable days missed from work per country was computed by multiplying employment rates with absenteeism rates from Eurostat 2022 [62] for France, Italy and Spain and ONS [63] for the UK, by the population size. The numbers were then multiplied by the average costs of each sick day.

| Table S2.15 Average wage per working day and per calendar day per full-time employee in euros. *CPI* Consumer price index; *OECD* Organisation for Economic Co-operation and Development; *UK* United Kingdom | | | | |
| --- | --- | --- | --- | --- |
| **Country** | **Annual gross wage** | **Per working day** | **Per calendar day** | **Sources** |
| France | €42,226 | €195 | €116 | OECD 2021 [61] and adjusted for CPI (OECD statistics) [53] |
| Italy | €32,105 | €145 | €88 |  |
| Spain | €29,767 | €131 | €82 |  |
| UK | €49,220 | €221 | €135 |  |
|  | | | | |

## Vaccine efficacy

The base case value relies on standard-dose quadrivalent inactivated vaccine (QIV-SD) vaccine efficacy which is derived from standard-dose trivalent inactivated vaccine (TIV-SD), vaccine efficacy meta-analyses in clinical trials from Cochrane reviews [65, 66, 67]. The base case value is adjusted for the benefit of having protection against both B lineages [64]. The TIV-SD vaccine efficacy by age group is presented in **Table S2.16**.

| **Table S2.16** TIV-SD vaccine efficacy against laboratory-confirmed influenza per age group from Cochrane reviews**.** *CI,* confidence interval*; QIV-SD* Standard-dose trivalent inactivated vaccine. Notes: VE corresponds to the reported value for inactivated influenza vaccine. Original CI were reported in terms of relative risk and hereby transformed to VE utilising its percental variation in a case-by-case basis for each risk group | | | |
| --- | --- | --- | --- |
|  | **Older adults (≥65 years)** | **6 months–17 years** | **18–65 years** |
| Vaccine efficacy | 58% | 64% | 59% |
| Upper bound of 95% CI | 91% | 85% | 68% |
| Lower bound of 95% CI | 37% | 49% | 52% |
| Sources | Demicheli et al. 2018 [65] | Jefferson et al. 2018 [66] | Demicheli et al. 2018 [67] |
|  | | | |

The following calculation accounts for B strain circulation, risk of mismatch, and the cross-protection:

$$\left( VE QIV \right)=\left( VE TIV \right)+\left( VE TIV \right)\times(\%B)\times(1-\%Bmatch)\times(1-Crossprotection)$$

*QIV* quadrivalent inactivated vaccine; *TIV* trivalent inactivated vaccine*; VE* vaccine efficacy

The equation considers the VE of TIV as the basis and adds the expected benefit of an additional B strain by weighting the core determinants of strain protection: a) incidence, b) match and c) cross-protection [68, 69].

Breaking the individual elements of the expected additional benefit of QIV:

- (%B) = Represents the participation of B strains (Victoria and Yamagata) among all circulating strains;
- (1-%B match) = The complement of the actual match of TIV’s selected B strain, that can also be interpreted as the observed mismatch related to the utilisation of a single B strain;
- (1-Cross Protection) = The complement of the known cross-protection of a given B strain, that can be seen as the gap to a full cross-protection.

This approach deductively uncovers the benefits of full B strain protection, by stepwise revealing the shortfalls of a single one.

A limitation of the formula involves the assumption that the efficacy against a given B strain is equivalent for the one, as individual differences cannot be captured.

The proportion of B strain circulation was estimated to be 25% on average in Europe, based on Caini et al. 2018 [70], 23% in the UK, reported by Ambrose et al. 2012 [71], 22% in Australia, reproted by Barr et al. 2012 [72], 7–25% reported in four countries in Europe/Asia (Turkey, Italy, Ukraine and England) by Caini et al. 2015 [73], and 17% reported in Australia by Moa et al. 2017 [74], who also stated that during 14 influenza seasons (2021–2014), B strain mismatches and partial matches occurred in 50% of the seasons. A 67% cross-protection was derived from mismatched seasons based on data reported by Diaz-Granados et al. 2012 [75].

The vaccine efficacy estimates and CIs used in the model are summarised in **Table S2.17**. Pepin et al. provides vaccine efficacy of QIV-SD against laboratory-confirmed cases for children 6–35 months in a Phase 3 trial [76]. The vaccine efficacy is 54.76% (40.24–66.03%) for participants aged 6–23 months and 46.91% (23.57–63.53%) for participants aged 24–35 months. These results provide an external validation of the proposed adjustment approach.

| Table S2.17 Estimated QIV-SD vaccine efficacy per age group. *CI* Confidence interval; *QIV-SD* Standard-dose quadrivalent inactivated vaccine | | | |  |
| --- | --- | --- | --- | --- |
|  | **Older adults (≥65 years)** | **6 months**–**17 years** | **18**–**65 years** | |
| Vaccine efficacy | 60% | 67% | 61% | |
| Upper bound of 95% CI | 95% | 89% | 71% | |
| Lower bound of 95% CI | 39% | 51% | 54% | |
|  | | | | |

The use of vaccine efficacy estimates from meta-analysis of randomised controlled trials in a Cochrane review was preferred, in order to base the input on the highest level of evidence. This input was critical in the model and was tested in the deterministic sensitivity analysis (DSA).

# 3. Model outputs

| **Table S3.1** Total influenza cases averted (%) at the 2021/2022 VCR, by risk group and country**.** *GP* General practitioner; *HCW* Healthcare worker; *UK* United Kingdom; *VCR* Vaccination coverage rate | | | | | | | | | |
| --- | --- | --- | --- | --- | --- | --- | --- | --- | --- |
| **Country** | **Children**  **(6–24 months)** | **Older adults**  **(≥65 years)** | **Individuals with chronic conditions  (2–17 years)** | **Individuals with chronic conditions (18–49 years)** | **Individuals with chronic conditions (50–64 years)** | **Pregnant Women** | **HCWs** | **Total** |  |
| France | 4,350 (1) | 349,050 (77) | 9,450 (2) | 33,950 (7) | 45,600 (10) | 1,400 (0) | 10,500 (2) | 454,300 |  |
| Italy | 3,600 (1) | 352,700 (82) | 4,950 (1) | 25,850 (6) | 39,050 (9) | 150 (0) | 4,600 (1) | 430,900 |  |
| Spain | 3,000 (1) | 285,600 (83) | 4,000 (1) | 14,700 (4) | 18,500 (5) | 4,400 (1) | 14,700 (4) | 344,900 |  |
| UK | 400 (0) | 445,700 (69) | 18,700 (3) | 65,600 (10) | 78,800 (12) | 6,000 (1) | 26,500 (4) | 641,700 |  |

# 4. Deterministic sensitivity analysis

To further analyse the public health benefits and economic impact of achieving 75% VCR for risk groups across France, Italy, Spain and the UK, a DSA was performed to explore the univariate change of the most sensitive parameters of the model shown in the **Table S4.1**.

| Table S4.1 DSA inputs and rationale. *CI* Confidence interval; *DSA* Deterministic sensitivity analysis; *GP* General practitioner; *HCW* Healthcare worker | | | |
| --- | --- | --- | --- |
| **Input** | **Low case** | **High case** | **Rationale** |
| Population size – Children  6–24 months | – | +20% | Arbitrary, to test a potential increase in birth cohorts |
| Population size – Individuals with chronic conditions | –30% | +40% | Sensitivity based on differences observed between Clark et al. [14] and official data from the UK [77] and France [78] |
| Population size – Older adults | – | +12.4% | Sensitivity based on ≥65 population size 2030 projections from Eurostat [79] |
| Population size – HCWs | –55% | +55% | Standard deviation based on the comparison between Eurostat HCW population size and national data from Germany [80], the UK [81], France [82], Spain [83] and Netherlands [84] |
| Population size – Pregnant women | –20% | +20% | ±20% arbitrary range |
| Vaccine Efficacy | Low boundary of CI | Upper boundary of CI | CI of the vaccine efficacy inputs |
| GP visit rate | –20% | +20% | ±20% range based on differences observed across seasons |
| GP visit rate – Positivity rate | Low boundary of CI | Upper boundary of CI | Acute respiratory infection/influenza-like illness factor CI from Belazi et al. [36] |
| GP visit rate – Risk factor | Without risk factor | – | Sensitivity based on the non-application of the 1.51 Baguelin et al. risk factor [35] |
| Hospitalisation rate | –20% | +20% | ±20% range based on differences observed across seasons |
| Hospitalisation rate – Risk factor | Without risk factor | – | Sensitivity based on the non-application of the 2.25 Coleman et al risk factor [44] |
| Absenteeism – Number of workdays lost conditional to GP visit | –20% | +20% | ±20% range justified by differences observed across seasons |
| Absenteeism – Number of workdays lost following hospital stay | Low boundary of CI | Upper boundary of CI | CI from Scholz et al. [50] |
| Absenteeism – Probability of taking sick leave – adults | 50% | 70% | Arbitrary sensitivity to test two levels of increase in the probability of taking sick leave |
| GP visit costs | –20% | +20% | ±20% arbitrary range, +20% to show the increase GP visit costs for an individual with chronic conditions |
| Hospitalisation costs | –20% | +20% | ±20% arbitrary range, +20 to show the increase hospitalisation costs for an individual with chronic conditions |
| Hospitalisation costs – Individuals with chronic conditions | – | +23% | Sensitivity based on the increase of hospitalisation costs for people with comorbidities during an influenza episode from Gil et al. [43] |
|  | | | |

#

# 5. Scenario analysis

A scenario with high dose (HD) QIV influenza vaccine for the older population was performed, because the Efluelda vaccine has been approved for adults older than 60 years old in 25 European countries, including France, Italy and Spain (though not the UK) [85]. Of note, this vaccine was preferentially recommended by the STIKO (Ständigen Impfkommission) in Germany for adults older than 60 years old [86]. A scenario with QIV-HD vaccine efficacy for the older population were therefore built for the countries in which this vaccine has been approved.

When using the 2021/22 QIV-HD vaccine efficacy for older adults for France, Italy and Spain, the number of cases averted by vaccinating the gap to 75% VCR would be 975,000 influenza cases, 340,000 GP visits, 18,000 hospital admissions, 7,500 deaths, €95 million of direct medical costs and €79 million of absenteeism costs (**Table S5.1**). A scenario with QIV-HD vaccine efficacy established that most public health cost benefits would be delivered in France with €88 million in total, followed by Spain with €45 million, Italy with €25 million and the UK with €15 million.

| Table S5.1 Public health events and costs averted by vaccinating the gap to 75% using QIV-HD vaccine efficacy for older adults. *GP* General practitioner; *QIV-HD* High-dose quadrivalent inactivated vaccine | | | |
| --- | --- | --- | --- |
| **Influenza-associated events averted** | **Number of events avoided** | **Costs avoided** |  |
| Cases | 975,000 | – |  |
| Deaths | 7,500 | – |  |
| GP visits | 340,000 | €13,351,500 |  |
| Hospitalisations | 18,000 | €81,444,800 |  |
| Lost days of work | 735,000 | €78,693,500 |  |
| All influenza-associated costs | – | €173,489,800 |  |
|  | | | |

Beyond the infection, influenza is a trigger for cardio-respiratory complications, which can lead to prolonged hospitalisations and medical support, and eventually death. Therefore, influenza-associated hospital admission rates were compared with those for excess influenza-associated hospitalisations for the three VCR levels (current VCR, 75% VCR and gap to 75% VCR) for France, Italy, Spain and the UK, focusing on the older adult population (≥65 years of age). Hospital admission rates for influenza only were obtained from national public health reports, while excess influenza-associated hospitalisation rates were the same as per the main model epidemiological inputs. Overall, the excess influenza-associated hospitalization rates were substantially higher than the influenza-only hospitalization rates in older adults, reinforcing the impact of influenza on cardio-respiratory complications and therefore the importance of a high VCR in older adults (Fig. S5.1).


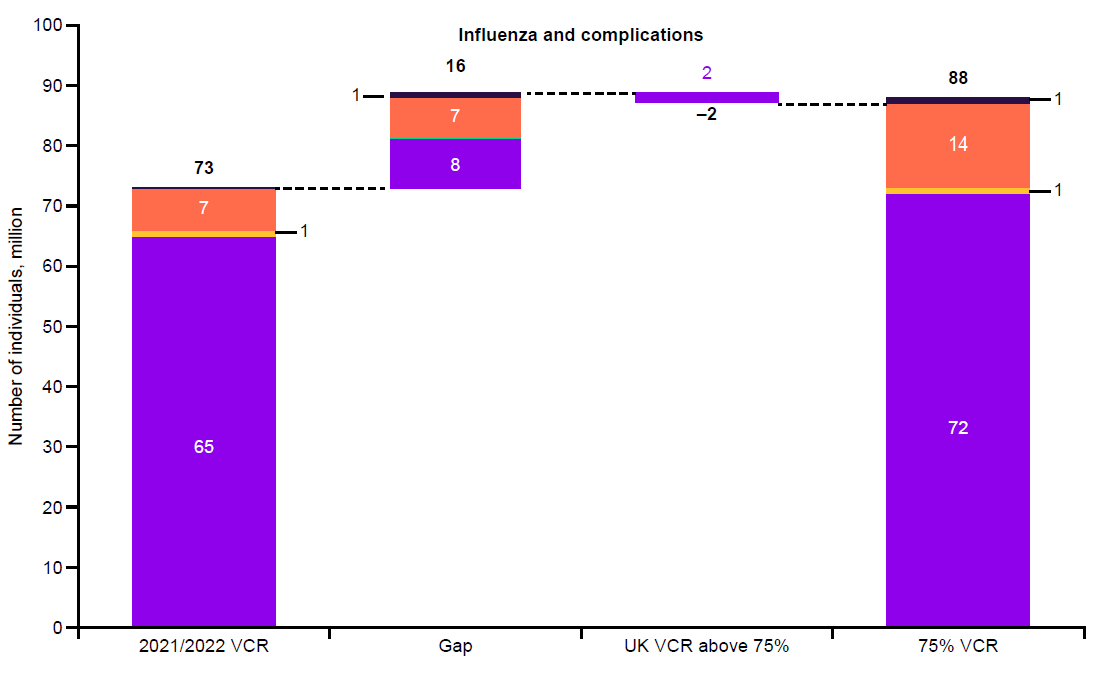
Fig. S5.1 Influenza and complications hospital admission rates versus influenza only hospital admission rates. *HCW* Healthcare workers; *UK* United Kingdom; *VCR* Vaccine coverage rate. Data are presented to the nearest integer; discrepancies in totals are due to rounding.

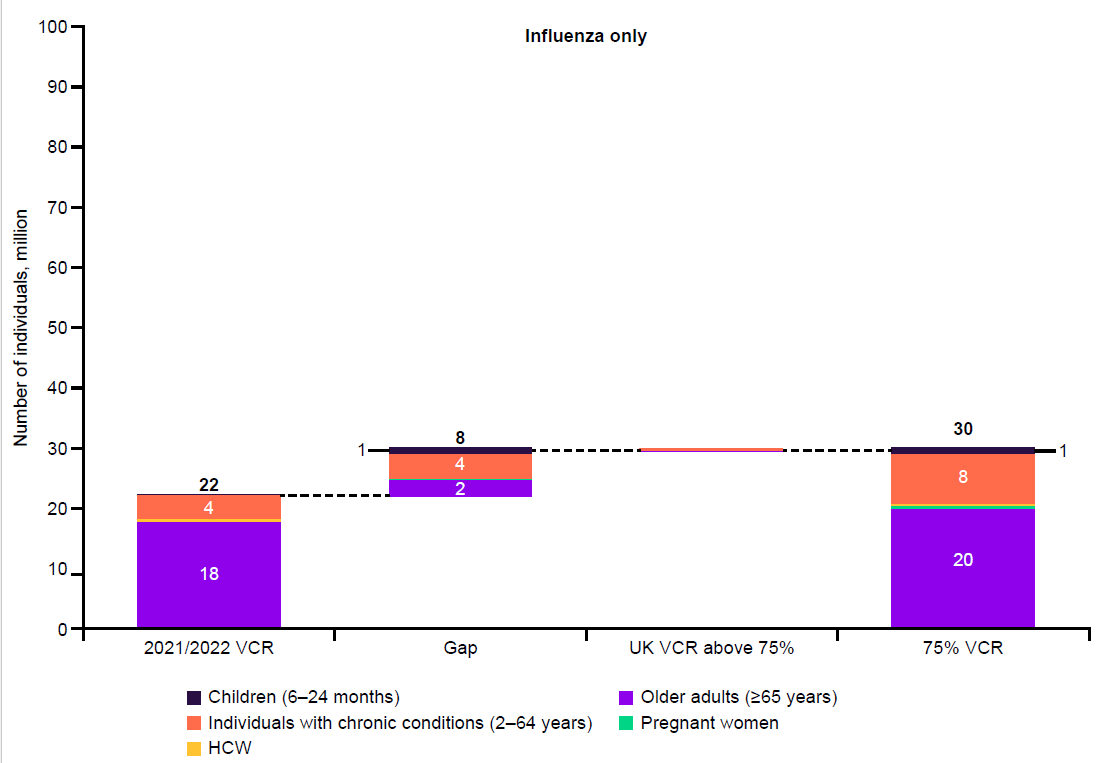


**6. Potential investment needed to achieve 75% VCR**

To illustrate the potential investment required to cover the additional gap to achieve 75% VCR in at-risk groups, Table S6.1 algebraically multiplies the average public acquisition price of QIV-SD by the number of individuals in the unvaccinated population. However, as the value of vaccination is best estimated in terms of long-term costs and quality-adjusted life years in the context of a willingness-to-pay threshold, these illustrative estimates should be interpreted cautiously. As the cost of vaccine acquisition is a single component of the resources necessary for implementation, with additional resources required for vaccine application and immunisation campaigns, estimated costs are unlikely to reflect the real value of vaccine acquisition for payors.

Table S6.1 Illustrative vaccine acquisition costs to cover the unvaccinated repository between current and 75% VCR (not exhaustive). HCW Healthcare worker; NA Not available,

VCR Vaccination coverage rate

| Country | Population | Number of individuals  needed to  achieve 75% VCR | Cost of vaccine | Total cost | Sources |
| --- | --- | --- | --- | --- | --- |
| France | Children  (6-24 months) | 731,506 | 11.75 € | € 8,595,196 | Base de donées publiques des médicaments 2024 [87] |
|  | Older adults | 2,589,111 |  | € 30,422,054 |  |
|  | Individuals with chronic conditions | 3,649,153 |  | € 42,877,548 |  |
|  | Pregnant woman | 435,412 |  | € 5,116,091 |  |
|  | HCWs | 940,516 |  | € 11,051,063 |  |
| Italy | Children (6-24 months) | 413,404 | 11.08 € | € 4,580,516 | Mennini et al. 2018 [88] |
|  | Older adults | 2,374,687 |  | € 26,311,532 |  |
|  | Individuals with chronic conditions | 3,995,157 |  | € 44,266,340 |  |
|  | Pregnant woman | 261,465 |  | € 2,897,032 |  |
|  | HCWs | 647,917 |  | € 7,178,920 |  |
| Spain | Children (6-24 months) | 350,440 | 9.5€ | € 4,380,500 | García et al. 2016 [89] |
|  | Older adults | 533,491 |  | € 6,668,638 |  |
|  | Individuals with chronic conditions | 4,221,714 |  | € 52,771,425 |  |
|  | Pregnant woman | 59,625 |  | € 745,313 |  |
|  | HCWs | 131,170 |  | € 1,639,625 |  |
| UK | Children (6-24 months) | 775,635 | 9.35 € | € 7,252,187 | British National Formulary 2024 [90] |
|  | Older adults | NA |  | NA |  |
|  | Individuals with chronic conditions | 2,338,474 |  | € 21,864,732 |  |
|  | Pregnant woman | 219,172 |  | € 2,049,258 |  |
|  | HCWs | 236,643 |  | € 2,212,612 |  |

# References

1. Higgins J, Green S. Cochrane Handbook for Systematic Reviews of Interventions, 5.1.0. The Cochrane Collaboration. 2011. <https://handbook-5-1.cochrane.org/>. Accessed 11 Jan 2024.
2. Santé publique France. <https://www.santepubliquefrance.fr/>. Accessed 14 Apr 2023.
3. Ministero della salute. <https://www.salute.gov.it/portale/influenza/dettaglioContenutiInfluenza.jsp?lingua=italiano&id=679&area=influenza&menu=vuoto>. Accessed 3 Apr 2023.
4. Ministerio de Sanidad. <https://www.sanidad.gob.es/>. Accessed 14 Apr 2023.
5. Public Health England. Seasonal influenza vaccine uptake in GP patients: winter season 2021 to 2022. [https://assets.publishing.service.gov.uk/government/uploads/system/uploads/attachment_data/file/1128172/GP-patients-flu-annual-report-2021-to-2022-corrected_final.pdf. Accessed 14 Apr 2023](https://assets.publishing.service.gov.uk/government/uploads/system/uploads/attachment_data/file/1128172/GP-patients-flu-annual-report-2021-to-2022-corrected_final.pdf.%20Accessed%2014%20Apr%202023).
6. European Centre for Disease Control and Prevention. Surveillance reports and disease data on seasonal influenza. <https://www.ecdc.europa.eu/en/seasonal-influenza/surveillance-reports-and-disease-data>. Accessed 11 Jan 2024.
7. European Commission. Influenza. <https://ec.europa.eu/health/vaccination/influenza_en>. Accessed 11 Jan 2024.
8. Eurostat. <https://ec.europa.eu/eurostat/fr/>. Accessed 11 Jan 2024.
9. A manual for estimating disease burden associated with seasonal influenza. <https://apps.who.int/iris/bitstream/handle/10665/178801/9789241549301_eng.pdf>. Accessed 11 Jan 2024.
10. WHO (World Health Organization) Seasonal influenza vaccination recommendations during the COVID-19 pandemic. Weekly Epidemiological Record. 2020;95:539–543. <https://apps.who.int/iris/bitstream/handle/10665/336260/WER9544-539-543-eng-fre.pdf?sequence=1&isAllowed=y>. Accessed 7 Jan 2024.
11. Eurostat. Total population. <https://ec.europa.eu/eurostat/databrowser/view/DEMO_PJAN__custom_5052874/default/table?lang=fr>. Accessed 20 Mar 2023.
12. Office for National Statistics. Estimates of the population for the UK, England, Wales, Scotland and Northern Ireland. <https://www.ons.gov.uk/peoplepopulationandcommunity/populationandmigration/populationestimates/datasets/populationestimatesforukenglandandwalesscotlandandnorthernireland>. Accessed 20 Mar 2023.
13. European Centre for Disease Prevention and Control (ECDC). Seasonal influenza vaccination and antiviral use in EU (European Union) / EEA (European Economic Area) Member States. <https://www.ecdc.europa.eu/en/publications-data/seasonal-influenza-vaccination-antiviral-use-eu-eea-member-states>. Accessed 11 Jan 2024.
14. Clark A, Jit M, Warren-Gash C, Guthrie B, Wang HH, Mercer S, et al. Global, regional, and national estimates of the population at increased risk of severe COVID-19 due to underlying health conditions in 2020: a modelling study. Lancet Glob Health. 2020;8:e1003–17.
15. Centers for Disease Control and Prevention (CDC). Similarities and differences between flu and COVID-19. <https://www.cdc.gov/flu/symptoms/flu-vs-covid19.htm>. Accessed 31 Oct 2023.
16. Eurostat. Live birth. <https://ec.europa.eu/eurostat/databrowser/view/DEMO_FMONTH__custom_5053091/default/table?lang=fr>. Accessed 20 Mar 2023.
17. Office for National Statistics. Births in England and Wales: summary tables. <https://www.ons.gov.uk/peoplepopulationandcommunity/birthsdeathsandmarriages/livebirths/datasets/birthsummarytables>. Accessed 20 Mar 2023.
18. National Records of Scotland. Birth, death and marriage Records. <https://www.nrscotland.gov.uk/research/guides/birth-death-and-marriage-records>. Accessed 20 Mar 2023.
19. Northern Ireland Statistics and Research Agency. Birth Statistics. <https://www.nisra.gov.uk/publications/birth-statistics>. Accessed 20 Mar 2023.
20. Eurostat. Self-reported vaccination against influenza by sex, age and degree of urbanisation. <https://ec.europa.eu/eurostat/databrowser/view/HLTH_EHIS_PA1U__custom_1209133/default/table?lang=en>. Accessed 20 Mar 2023.
21. Organisation for Economic Co-operation and Development (OECD). Healthcare workers population. <https://stats.oecd.org/Index.aspx?ThemeTreeId=9>. Accessed 20 Mar 2023.
22. European Centre for Disease Control and Prevention. Seasonal influenza vaccination and antiviral use in EU/EEA member states. <https://ecdc.europa.eu/en/publications-data/seasonal-influenza-vaccination-antiviral-use-eu-eea-member-states>. Accessed 11 Oct 2023.
23. Vaux S, Van Cauteren D, Guthmann JP, Le Strat Y, Vaillant V, De Valk H, et al. Influenza vaccination coverage against seasonal and pandemic influenza and their determinants in France: A cross-sectional survey. BMC Public Health. 2011;11:30.
24. Lopez-de-Andres A, Hernández-Barrera V, Carrasco-Garrido P, Gil-de-Miguel Á, Jiménez-García R. Influenza vaccination coverage among Spanish children, 2006. Public Health. 2009;123(7):465-9.
25. Santé publique France. Données de couverture vaccinale grippe par groupe d’âge. <https://www.santepubliquefrance.fr/determinants-de-sante/vaccination/articles/donnees-de-couverture-vaccinale-grippe-par-groupe-d-age>. Accessed 3 Apr 2023.
26. Comité Asesor de Vacunas de la AEP. Coberturas de la vacunación antigripal en la temporada 2021-22. <https://vacunasaep.org/profesionales/noticias/gripe-coberturas-vacunacion-temporada-2021-22>. Accessed 3 Apr 2023.
27. Haute autorité de santé. Vaccination contre la grippe 2022-2023. <https://www.has-sante.fr/jcms/p_3345196/fr/avis-n2022-0036/ac/sespev-du-16-juin-2022-du-college-de-la-haute-autorite-de-sante-relatif-au-lancement-de-la-campagne-de-vaccination-2022/2023-contre-la-grippe-saisonniere-en-france-dans-l-hemisphere-nord-et-a-mayotte-dans-le-contexte-de-l-epidemie-de-covid-19>. Accessed 3 Apr 2023
28. Bianchi FP, Stefanizzi P, Cuscianna E, Di Lorenzo A, Migliore G, Tafuri S, et al. Influenza vaccine coverage in 6months-64 years-old patients affected by chronic diseases: A retrospective cohort study in Italy. Hum Vaccin Immunother. 2023;19(1):2162301.
29. Mochales JA. Coberturas de vacunación antigripal. Análisis de registros informaticos individualizados y encuestas poblacionales. Coberturas de vacunación antigripal Análisis de registros informaticos individualizados y encuestas poblacionales. 2017. <https://www.educacion.gob.es/teseo/imprimirFicheroTesis.do?idFichero=RGbJ9MFPAgg%3D>. Accessed 30 Oct 2023.
30. United Kingdom Government. Seasonal flu and COVID-19 vaccine uptake in frontline healthcare workers: monthly data, 2021 to 2022. <https://www.gov.uk/government/statistics/seasonal-flu-and-covid-19-vaccine-uptake-in-frontline-healthcare-workers-monthly-data-2021-to-2022>. Accessed 3 Apr 2023.
31. Somes MP, Turner RM, Dwyer LJ, Newall AT. Estimating the annual attack rate of seasonal influenza among unvaccinated individuals: a systematic review and meta-analysis. Vaccine. 2018;36:3199–207.
32. Wells KB, Miranda J, Bauer MS, Bruce ML, Durham M, Escobar J, et al. Overcoming barriers to reducing the burden of affective disorders. Biol Psychiatry. 2002;52(6):655–75.
33. Instituto de Salud Carlos III (ISCIII). Informes anuales. <https://www.isciii.es/QueHacemos/Servicios/VigilanciaSaludPublicaRENAVE/EnfermedadesTransmisibles/Paginas/Informes-anuales.aspx>. Accessed 30 Jan 2024.
34. Lemaitre M, Fouad F, Carrat F, Crépey P, Gaillat J, Gavazzi G, et al. Estimating the burden of influenza-related and associated hospitalizations and deaths in France: An eight-season data study, 2010–2018. Influenza and Other Respiratory Viruses. 2022;16:717–25.
35. Baguelin M, Camacho A, Flasche S, Edmunds WJ. Extending the elderly and risk-group programme of vaccination against seasonal influenza in England and Wales: a cost-effectiveness study. BMC Med. 2015;13:236.
36. Belazi S, Olsen SJ, Brown C, Green HK, Mook P, Nguyen-Van-Tam J, et al. Spotlight influenza: Laboratory-confirmed seasonal influenza in people with acute respiratory illness: a literature review and meta-analysis, WHO European Region, 2004 to 2017. Euro Surveill. 2021;26(39):pii=2000343.
37. Carrat F, Sahler C, Rogez S, Leruez-Ville M, Freymuth F, Le Gales C, et al. Influenza burden of illness: Estimates from a national prospective survey of household contacts in France. Arch Intern Med. 2002;162(16):1842‒8.
38. Paternoster M, Masse S, van der Werf S, Lina B, Levy-Bruhl D, Villechenaud N, et al. Estimation of influenza-attributable burden in primary care from season 2014/2015 to 2018/2019, France. Eur J Clin Microbiol Infect Dis. 2021;40(6):1263‒9.
39. Mennini FS, Bini C, Marcellusi A, Rinaldi A, Franco E. Cost-effectiveness of switching from trivalent to quadrivalent inactivated influenza vaccines for the at-risk population in Italy. Hum Vaccin Immunother. 2018;14(8):1867‒73.
40. Fleming DM, Taylor RJ, Haguinet F, Schuck-Paim C, Logie J, Webb DJ, et al. Influenza-attributable burden in United Kingdom primary care. Epidemiol Infect. 2016;144(3):537‒47.
41. Bertolani A, Fattore G, Pregliasco F. The hospitalization burden of influenza: just the tip of the iceberg? Global & Regional Health Technology Assessment: Italian; Northern Europe and Spanish. Global & Regional Health Technology Assessment. 2018;2018:1‒9.
42. Matias G, Taylor RJ, Haguinet F, Schuck-Paim C, Lustig RL, Fleming DM. Modelling estimates of age-specific influenza-related hospitalisation and mortality in the United Kingdom. BMC Public Health. 2016;16:481.
43. Gil A, Díez-Domingo J, Martinón-Torres F, Redondo E, Ortiz de Lejarazu R, Correia de Sousa J, et al. Burden of severe influenza in the Iberian Peninsula: An administrative database analysis over 10 influenza seasons (2008/09-2017/18). ESWI poster. 2021.
44. Coleman BL, Fadel SA, Fitzpatrick T, Thomas SM. Risk factors for serious outcomes associated with influenza illness in high- versus low- and middle-income countries: Systematic literature review and meta-analysis. Influenza Other Respir Viruses. 2018;12:22–29.
45. Preaud E, Durand L, Macabeo B, Farkas N, Sloesen B, Palache A, et al. Annual public health and economic benefits of seasonal influenza vaccination: A European estimate. BMC Public Health. 2014;14:813.
46. Rosano A, Bella A, Gesualdo F, Acampora A, Pezzotti P, Marchetti S, et al. Investigating the impact of influenza on excess mortality in all ages in Italy during recent seasons (2013/14–2016/17 seasons). International Journal of Infectious Diseases. 2019;88:127–34.
47. Gil A, Díez-Domingo J, Martinón-Torres F, Redondo E, Ortiz de Lejarazu R, Correia de Sousa J, et al. Burden of severe influenza in the Iberian Peninsula: An excess modelling analysis of hospitalization and mortality attributable to influenza over 10 epidemic seasons (2008/09-2017/18). ESWI poster. 2021.
48. Public Health England. Surveillance of influenza and other respiratory viruses in the UK. <https://www.gov.uk/government/statistics/annual-flu-reports>. Accessed 20 Mar 2023
49. Nielsen J, Vestergaard LS, Richter L, Schmid D, Bustos N, Asikainen T, et al. European all-cause excess and influenza-attributable mortality in the 2017/18 season: should the burden of influenza B be reconsidered? Clinical Microbiology and Infection. 2019;25(10):1266‒76.
50. Scholz S, Damm O, Schneider U, Ultsch B, Wichmann O, Greiner W. Epidemiology and cost of seasonal influenza in Germany - a claims data analysis. BMC Public Health. 2019;19(1):1090.
51. Diel R, Nienhaus A. Cost–benefit analysis of real-time influenza testing for patients in german emergency rooms. Int J Environ Res Public Health. 2019;16(13):2368.
52. Gerlier L, Lamotte M, Grenèche S , Lenne X, Carrat F, Weil-Olivier C, et al. Assessment of Public Health and Economic Impact of Intranasal Live-Attenuated Influenza Vaccination of Children in France Using a Dynamic Transmission Model. Appl Health Econ Health Policy. 2017;15(2):261–76.
53. Organisation for Economic Co-operation and Development (OECD). Inflation (CPI). <https://data.oecd.org/price/inflation-cpi.htm>. Accessed 11 Jan 2024.
54. European Central Bank (ECB). Statistical Data Warehouse, Exchange Rates. <https://sdw.ecb.europa.eu/browseSelection.do?node=1495>. Accessed 27 Oct 2021.
55. Améli. Consultations et actes : Les tarifs conventionnels. <https://www.ameli.fr/medecin/exercice-liberal/remuneration/consultations-actes/tarifs>. Accessed 15 Jan 2024.
56. Capri S, Barbieri M, de Waure C, Boccalini S, Panatto D. Cost-effectiveness analysis of different seasonal influenza vaccines in the elderly Italian population. Hum Vaccin Immunother. 2018;14(6):1331‒41.
57. Hodgson D, Baguelin M, van Leeuwen E, Panovska-Griffiths J, Ramsay M, Pebody R, et al. Effect of mass paediatric influenza vaccination on existing influenza vaccination programmes in England and Wales: a modelling and cost-effectiveness analysis. Lancet Public Health. 2017;2(2):e74–81.
58. Boccalini S, Bechini A, Innocenti M, Sartor G, Manzi F, Bonanni P, et al. La vaccinazione universale dei bambini contro l’influenza con il vaccino Vaxigrip Tetra® in Italia: risultati di una valutazione di Health Technology Assessment (HTA). J Prev Med Hyg. 2018;59:1:E1‒86.
59. Dal Negro RW, Turco P, Povero M. Cost of influenza and influenza-like syndromes (I-LSs) in Italy: Results of a cross-sectional telephone survey on a representative sample of general population. Respir Med. 2018;141:144‒9.
60. Moss JWE, Davidson C, Mattock R, Gibbons I, Mealing S, Carroll S. Quantifying the direct secondary health care cost of seasonal influenza in England. BMC Public Health. 2020;20:1464.
61. Organisation for Economic Co-operation and Development (OECD). Average annual wages. <https://stats.oecd.org/viewhtml.aspx?datasetcode=AV_AN_WAGE&lang=en>. Accessed 11 Jan 2024.
62. Eurostat. Employment and activity by sex and age, annual data. <https://ec.europa.eu/eurostat/databrowser/view/LFSI_EMP_A/default/table?lang=en>. Accessed 30 Mar 2023.
63. Office for National Statistics. A02 SA: Employment, unemployment and economic inactivity for people aged 16 and over and aged from 16 to 64 (seasonally adjusted). <https://www.ons.gov.uk/employmentandlabourmarket/peopleinwork/employmentandemployeetypes/datasets/employmentunemploymentandeconomicinactivityforpeopleaged16andoverandagedfrom16to64seasonallyadjusteda02sa>. Accessed 14 Apr 2023.
64. Jefferson T, Di Pietrantonj C, Rivetti A, Bawazeer G, Al-Ansary L, Ferroni E. Vaccines for preventing influenza in healthy adults. Cochrane Database Syst Rev. 2010;(7):CD001269.
65. Demicheli V, Jefferson T, Di Pietrantonj C, Ferroni E, Thorning S, Thomas RE, et al. Vaccines for preventing influenza in the elderly. Cochrane Database Syst Rev. 2018;2(2):CD004876.
66. Jefferson T, Rivetti A, Di Pietrantonj C, Demicheli V. Vaccines for preventing influenza in healthy children. Cochrane Database Syst Rev. 2018;2(2):CD004879.
67. Demicheli V, Jefferson T, Ferroni E, Rivetti A, Di Pietrantonj C. Vaccines for preventing influenza in healthy adults. Cochrane Database Syst Rev. 2018;2(2):CD001269.
68. Choi EJ, Park JH, Chun BC. Cost effectiveness of trivalent and quadrivalent influenza vaccines in 50- to 64-year-old adults in Korea. Vaccine. 2020;38(32):5002‒8.
69. Kittikraisak W, Chittaganpitch M, Gregory CJ, Laosiritaworn Y, Thantithaveewat T, Dawood FS, et al. Assessment of potential public health impact of a quadrivalent inactivated influenza vaccine in Thailand. Influenza Other Respir Viruses. 2016;10(3):211‒19.
70. Caini S, Kroneman M, Wiegers T, El Guerche-Séblain C, Paget J. Clinical characteristics and severity of influenza infections by virus type, subtype, and lineage: A systematic literature review. Influenza Other Respir Viruses. 2018;12(6):780‒92.
71. Ambrose CS, Levin MJ. The rationale for quadrivalent influenza vaccines. Hum Vaccin Immunother. 2012;8(1):81‒8.
72. Barr IG, Jelley LL. The coming era of quadrivalent human influenza vaccines: who will benefit? Drugs. 2012;72(17):2177‒85.
73. Caini S, Huang QS, Ciblak MA, Kusznierz G, Owen R, Wangchuk S, et al. Epidemiological and virological characteristics of influenza B: results of the Global Influenza B Study. Influenza Other Respir Viruses. 2015;9 Suppl 1(Suppl 1):3‒12.
74. Moa AM, Muscatello DJ, Turner RM, MacIntyre CR. Epidemiology of influenza B in Australia: 2001‒2014 influenza seasons. Influenza Other Respir Viruses. 2017;11(2):102‒9.
75. DiazGranados CA, Denis M, Plotkin S. Seasonal influenza vaccine efficacy and its determinants in children and non-elderly adults: a systematic review with meta-analyses of controlled trials. Vaccine. 2012;31(1):49‒57.
76. Pepin S, Samson SI, Alvarez FP, Dupuy M, Gresset-Bourgeois V, De Bruijn I. Impact of a quadrivalent inactivated influenza vaccine on influenza-associated complications and health care use in children aged 6 to 35 months: Analysis of data from a phase III trial in the Northern and Southern Hemispheres. Vaccine. 2019;37(13):1885‒8.
77. Office for National Statistics. People with long-term health conditions, UK: January to December 2019. <https://www.ons.gov.uk/peoplepopulationandcommunity/healthandsocialcare/conditionsanddiseases/adhocs/11478peoplewithlongtermhealthconditionsukjanuarytodecember2019>. Accessed 14 Apr 2023.
78. Insee. État de santé de la population. https://www.insee.fr/fr/statistiques/4238405?sommaire=4238781. Accessed 14 Apr 2023.
79. Eurostat. Population on 1st January by age, sex and type of projection. <https://ec.europa.eu/eurostat/databrowser/view/PROJ_19NP__custom_1878410/default/line?lang=en>. Accessed 14 Apr 2023.
80. German Federal Statistical Office. Health personnel. <https://www.destatis.de/EN/Themes/Society-Environment/Health/Health-Personnel/_node.html>. Accessed 14 Apr 2023.
81. National Health Service (NHS) England. Healthcare Worker Reporting FAQs. <https://www.england.nhs.uk/south/wp-content/uploads/sites/6/2022/08/Reporting-FAQs-Jul22-v1FINAL.pdf>. Accessed 14 Apr 2023.
82. Insee. Personnels et équipements de santé. <https://www.insee.fr/fr/statistiques/4277748?sommaire=4318291>. Accessed 14 Apr 2023.
83. Ministerio de Sanidad. Vacunas y Programa de Vacunación. <https://www.sanidad.gob.es/profesionales/saludPublica/prevPromocion/vacunaciones/calendario-y-coberturas/coberturas/home.htm>. Accessed 14 Apr 2023.
84. BIG-register. Cijfers, over het BIG-register. <https://www.bigregister.nl/over-het-big-register/cijfers>. Accessed 14 Apr 2023.
85. Sanofi. Sanofi va construire une nouvelle installation au Canada pour accroître la disponibilité mondiale de son vaccin à haute dose contre la grippe. <https://www.sanofi.com/fr/media-room/communiques-de-presse/2021/2021-03-31-14-05-00-2202566>. Accessed 14 Apr 2023.
86. Robert Koch-Institut (RKI). Epidemiologisches Bulletin 2021. <https://www.rki.de/DE/Content/Infekt/EpidBull/Archiv/2021/01/Tabelle.html;jsessionid=24A230AD6414D945791E39A4474D7164.internet071?nn=2371176https://www.rki.de/DE/Content/Infekt/EpidBull/Archiv/2021/01/Tabelle.html;jsessionid=24A230AD6414D945791E39A4474D7164.internet071?nn=2371176>. Accessed 27 Jan 2024.
87. Base de donées publiques des médicaments. VAXIGRIPTETRA, suspension injectable en seringue préremplie. Vaccin grippal quadrivalent (inactivé, à virion fragmenté). Accessed 12 April 2024.
88. Mennini FS, Bini C, Marcellusi A, Rinaldi A, Franco E. Cost-effectiveness of switching from trivalent to quadrivalent inactivated influenza vaccines for the at-risk population in Italy. Hum Vaccin Immunother. 2018;14(8):1867–73.
89. García A, Ortiz de Lejarazu R, Reina J, Callejo D, Cuervo J, Morano Larragueta R. Cost-effectiveness analysis of quadrivalent influenza vaccine in Spain. Hum Vaccin Immunother. 2016;12(9):2269–77.
90. Medicines complete. British National Formulary 2024. https://www.medicinescomplete.com/#/content/bnf/_118509221?hspl=influenza#DMD34680411000001107. Accessed 12 April 2024.
