# Supplementary material for: Public health and economic benefits of seasonal influenza vaccination in risk groups in France, Italy, Spain and the UK: state of play and perspectives
Source: BMC Public Health. 2024 May 3;24:1222. doi: 10.1186/s12889-024-18694-5 (PMC11067100; doi:10.1186/s12889-024-18694-5)
Supplement: Supplementary file 1 — Supplementary material 1. [file 12889_2024_18694_MOESM1_ESM.zip › Supp Figure 1b.pdf]

B Hierarchy of included studies

Economic burden – Hierarchy of included studies

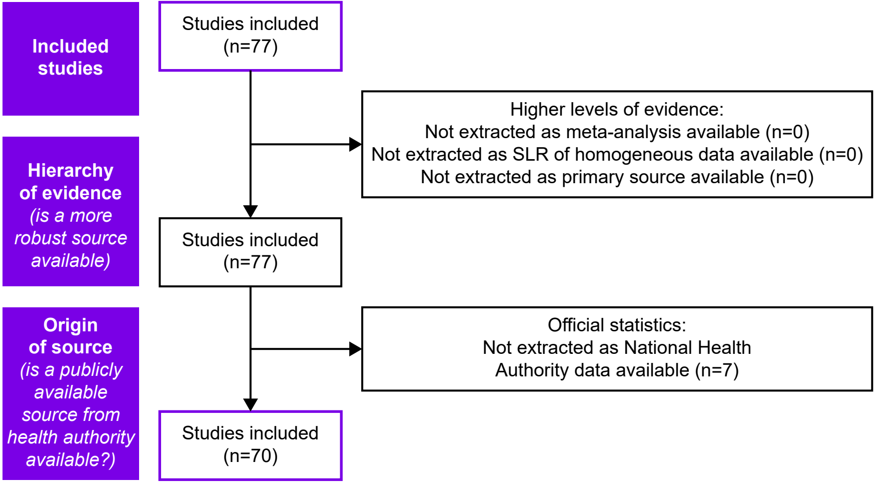

Hierarchy of sources:

- Preferred outcome (only for Epi data - cf detailed slide)
- **OCEBM** levels of evidence (meta-analysis, SLR, RCTs, cohort studies, case control studies, ecological, ...)
- **Origin of source** (e.g. official statistics vs publication etc...)
