# Supplementary material for: Public health and economic benefits of seasonal influenza vaccination in risk groups in France, Italy, Spain and the UK: state of play and perspectives
Source: BMC Public Health. 2024 May 3;24:1222. doi: 10.1186/s12889-024-18694-5 (PMC11067100; doi:10.1186/s12889-024-18694-5)
Supplement: Supplementary file 1 — Supplementary material 1. [file 12889_2024_18694_MOESM1_ESM.zip › Supp Figure 1c.pdf]

C Data extraction decision

Economic burden – Extraction decision tree

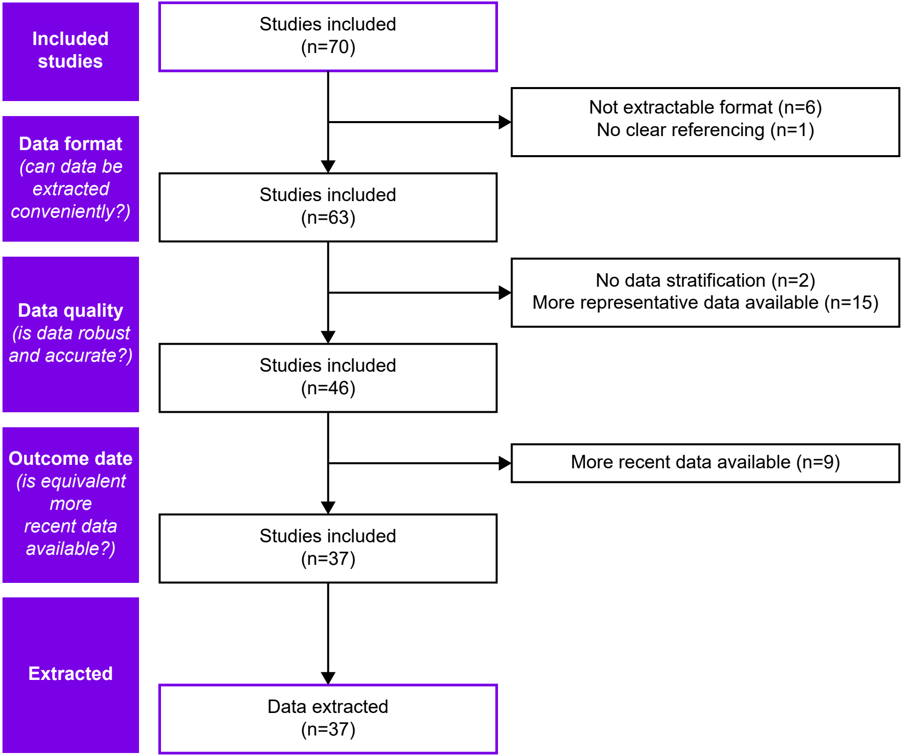

Final decision on data extraction

based on (for all other parameters equal):

- Data format
- Data representativeness/quality
- Most recent data
